# Genetics of retroactive measures of stress response in pigs before and after exposure to a disease challenge

**DOI:** 10.1093/g3journal/jkag005

**Published:** 2026-01-13

**Authors:** Fazhir Kayondo, Hayder Al-Shanoon, Yolande M Seddon, Dylan Carette, Carmen Cole, David M Janz, Frederic Fortin, John C S Harding, Michael K Dyck, Graham S Plastow, Pig Gen Canada, Jack C M Dekkers

**Affiliations:** Department of Animal Science, Iowa State University, Ames, IA 50011, United States; Western College of Veterinary Medicine, University of Saskatchewan, Saskatoon, SK S7N 5B4, Canada; Western College of Veterinary Medicine, University of Saskatchewan, Saskatoon, SK S7N 5B4, Canada; Western College of Veterinary Medicine, University of Saskatchewan, Saskatoon, SK S7N 5B4, Canada; Western College of Veterinary Medicine, University of Saskatchewan, Saskatoon, SK S7N 5B4, Canada; Western College of Veterinary Medicine, University of Saskatchewan, Saskatoon, SK S7N 5B4, Canada; Centre de Devéloppement du Porc du Québec Inc., Québec City, QC G1V 4M6, Canada; Western College of Veterinary Medicine, University of Saskatchewan, Saskatoon, SK S7N 5B4, Canada; Department of Agricultural, Food and Nutritional Science, University of Alberta, Edmonton, AB T6G 2R3, Canada; Department of Agricultural, Food and Nutritional Science, University of Alberta, Edmonton, AB T6G 2R3, Canada; PigGen Canada Research Consortium, Guelph, ON N1H4G8, Canada; Department of Animal Science, Iowa State University, Ames, IA 50011, United States

**Keywords:** stress hormones, genetics, pigs, hair

## Abstract

This study explored the genetics of cortisol (CL), cortisone (CN), DHEA (DH), and DHEA-S (DS) in hair of 610 pigs that were grown while they were exposed to infectious stressors (IS) from a natural polymicrobial disease challenge. Results were then contrasted with previous results on hair from these same pigs grown while experiencing noninfectious stressors (NIS), such as weaning, castration, transportation, and mixing. All pigs were genotyped for 50 K SNPs and imputed to 650 K SNPs. Heritability estimates for hormone levels in hair grown under IS ranged from 0.01 for DS to 0.27 for CL. Estimates of genetic correlations between levels of a hormone in hair grown in response to IS vs NIS were not significantly different from zero and were highest, at 0.52, for CL. Genome-wide association studies identified the same major QTL for CL in response to IS that was previously found for response to NIS, near the glucocorticoid receptor gene. The minor allele at the lead SNPs (frequency = 9%) significantly (*P* < 0.001) reduced CL under IS by 30 ± 4% and CN by 23 ± 6%, had no significant effect on DH or DS, and drove the genetic correlation between CL in hair grown under NIS vs IS. A comparative gene set enrichment analysis (GSEA) approach revealed that genomic windows that were associated with active forms of the stress hormones (CL and DH) tended to explain more variance during response to IS than to NIS, while the opposite was true for their inactive forms (CN and DS). These results may facilitate the selection of pigs that cope better with IS and NIS using hormone levels in hair as a noninvasive sample.

## Introduction

In commercial production, pigs face multiple noninfectious stressors (NIS) such as weaning, transportation, painful procedures at processing, social mixing, and human handling, as well as infectious stressors (IS) in the form of disease. Animals respond both physically and physiologically to any form of stress to restore homeostasis (Chu et al. 2024). Physiologically, stress response is mediated through co-operation of the nervous, endocrine, immune, and other physiological systems and involves activation of the sympathetic-adreno-medullar axis, the hypothalamic–pituitary–adrenal (HPA) axis, and the immune system (Mifsud and Reul 2018). Activation of the HPA axis results in the production of cortisol (CL), which can be converted to cortisone (CN), and in the production of dehydroepiandrosterone (DHEA, DH), which can be sulfated to its storage form (DHEA-S, DS). These hormones interact to modulate stress responses and to restore physiological homeostasis (Tsigos et al. 2020).

The levels of stress hormones in hair of pigs provide a retroactive assessment of stress responses that occurred over the period of hair growth. Our previous research showed that the levels of stress hormones in hair of young and clinically healthy pigs, which accumulated primarily in response to NIS such as weaning, transportation, and mixing, are heritable (Kayondo et al. 2025) and that CL was genetically correlated with the number and intensity of vocalizations during a backtest. These results suggest that the level of cortisol in hair of young healthy pigs can be a genetic indicator of the pig's innate coping style to NIS. However, pigs in commercial environments also face IS, in addition to the NIS that are mostly part of routine operations.

Studies have shown that the HPA axis is activated during response to NIS, resulting in the production of stress hormones (Morgan et al. 2009; Mormede and Terenina 2012) and also during response to IS (DeMorrow 2018). We thus hypothesized that similar physiological processes are involved in a pig's response to IS and NIS, such that stress hormone levels in hair grown during response to NIS may be genetically correlated with levels of these stress hormones in hair grown during response to IS. In addition, given our previous work that levels of stress hormones in hair grown during response to NIS are heritable, we hypothesized that stress hormones in hair grown during response to IS are also heritable.

This study, therefore, aimed to determine the genetic basis of the levels of stress hormones that accumulated in hair of pigs grown during their exposure to a natural polymicrobial disease challenge, as well as their phenotypic and genetic correlations with the levels of stress hormones in hair of clinically healthy pigs, grown during exposure to NIS. We also evaluated the genetic and phenotypic correlations of stress hormone levels in hair with responses to a standard backtest, which is used to evaluate the coping style response of pigs to NIS (Hessing et al. 1993). We further performed genome-wide association studies (GWAS) to identify and characterize genomic regions that are associated with stress hormone levels in hair of pigs grown under disease and how these differ from the results reported by Kayondo et al. (2025) for stress hormone levels in hair grown during response to NIS.

## Materials and methods

### Animals used

Fifteen batches of 60 or 75 Yorkshire × Landrace barrows, each from one high-health multiplier farm from one of seven breeding companies, members of PigGen Canada (Alliance Genetics, AlphaGene, DNA Genetics, AcuFast, Genesus, Hypor, and Topigs-Norsvin), were transported to a healthy quarantine nursery facility (qNur) in Quebec, Canada, at ∼21 d of age. A batch was entered into the quarantine nursery every 3 weeks from one of the 7 companies, in rotation, and acclimated for 3 weeks before being moved to the natural disease challenge nursery (cNur), where pigs were exposed to common swine pathogens in a natural polymicrobial disease challenge through contact for 1 week with the previous batch in a continuous flow system, as detailed by Putz et al. (2019) and Cheng et al. (2020). After ∼27 d in the challenge nursery, pigs were moved to the finisher, which was in the same barn and shared airspace with the challenge nursery. The disease challenge was designed to maximize expression of differences in disease resilience but with careful veterinary oversight and control, as specified in Putz et al. (2019) and Cheng et al. (2020). Pathogens present were described by Putz et al. (2019).

### Genotypes

All pigs in these 15 batches were genotyped using a custom 50 K Affymetrix SNP panel. We then used the 650 K genotypes of the 50 earlier batches described by Cheng et al. (2020), which consisted of animals from the same companies and lines, to impute the 50 K SNPs to the 650 K panel using FImpute (Sargolzaei et al. 2014). Imputation was performed separately for each company. After quality control, we retained genotypes on 451,343 SNPs that mapped to the 19 chromosomes of the *Sus scrofa* genome. Markers on the X chromosome were included and coded as homozygotes for the part of the X chromosome that is not homologous to Y.

### Phenotypes

#### Backtest responses

The backtest was performed on 889 pigs at about 27 d of age, as described by Kayondo et al. (2025). Briefly, each pig was placed in a supine position in a V-shaped restrainer that was placed on a panel on the top of the adjacent pen, with the tail toward the pen and the head toward the wall. Upon restraint, the number and intensity of vocalizations (VN and VI) and of struggles (SN and SI) were recorded separately by two trained technicians for 30 s, with the same technician scoring all pigs in a batch for a given trait. The effect of the technicians was, therefore, confounded with the effect of batch.

#### Stress hormone levels

Levels of CL, CN, DH, and DS were measured in hair shaved at about 40 d of age from the hip area of 863 pigs, shortly before they left the quarantine nursery (qNur samples), as described by Kayondo et al. (2025). The same procedures were used to measure levels of these hormones in regrown hair at about 82 d of age, after they exited the challenge nursery (cNur samples). Due to mortality during the disease challenge, only 610 pigs provided cNur samples. In addition, some pigs lacked data for CN and DS due to a small amount of hair that was available after the assays for the active forms of the hormones (CL and DH) that were given priority. We tested whether missing of hormone data in cNur was associated with the level of hormones in the qNur samples via a log-likelihood test on models with and without the fixed effect of a pig having different combinations of missing hormone data in cNur (7 levels). This effect was not significant for any of the four hormones (CL, *P* = 0.42; CN, *P* = 0.71; DH, *P* = 0.95; DS, *P* = 0.84). The same analysis was performed to assess the association of missing hormone levels in cNur with the performance and resilience data that were collected before collection of the second hair sample and none of these were significant either (*P* > 0.05). There was, thus, no statistical evidence that pigs lacking hormone data in cNur were a nonrandom sample of the pigs with data up to that point.

The cNur samples for CL, DH, and DS were assayed in duplicate and final assay results for each pig were obtained as the average of these. For CN, samples were assayed in triplicate but, for reasons described in Kayondo et al. (2025), the final value used was the average of two replicates that resulted in the lowest coefficient of variation (CV). As a general quality control step, samples with a CV > 20% between replicates were reanalyzed and the new values were used if they had a relatively lower CV. Following this criterion, assays for 44, 62, and 24 cNur samples were reanalyzed for CL, CN, and DH, respectively. Of these, 4 samples for CL and 2 samples for DH still had a high CV (>20%) upon reanalysis. Because statistical analyses with or without these samples did not affect the estimates of the variance components, they were retained in the final dataset. Additionally, visual inspection of the distributions of the average of replicates identified 5 outlier samples with DS > 35.3 pg/mg and 1 sample with DH > 151.2 pg/mg, and these were eliminated from the dataset. Quality control procedures and removal of outliers for the qNur samples were described by Kayondo et al. (2025) but followed similar procedures.

#### Statistical analyses

Due to the skewness of the distribution of the hormone levels and their ratios, natural log-transformed values were used in the analyses. Ratios evaluated included CL/CN, CL/DH, CL/DS, CN/DH, CN/DS, DH/DS, and the ratio of the sum of glucocorticoids (SOGs = CL + CN) to the sum of DHEA(S) (SOD = DH + DS). Levels of CN and DS (inactive hormones) tended to have different concentrations depending on the length of storage in ground form before hormone extraction (see Supplementary Fig. 1). Hence the length (days) of storage was included in the models for hormone traits as a fixed covariate.

#### Estimation of genetic parameters

Genetic parameters for cNur stress hormone levels were estimated using the same general mixed linear model that was used for qNur stress hormone levels by Kayondo et al. (2025), with ASReml 4.2 (Gilmour et al. 2021). Fixed effects included batch and the covariates of age at entry into the quarantine nursery and, for stress hormone levels, length of storage (in days) of the ground hair samples before hormone extraction. Random effects included animal additive genetics, pen, litter (only for qNur hormone levels and for VI and SI), and residuals. Litter effects did not explain a substantial proportion of the phenotypic variance (data not shown) and were thus eliminated from models for hormone traits measured in cNur.

Bivariate analyses were used to estimate genetic (${\hat{r}}_{g}$) and phenotypic (${\hat{r}}_{p}$) correlations by linear mixed models. For VN and SN, ln(1 + count) was used in their bivariate analyses, following Kayondo et al. (2025). Based on estimates from univariate analyses, the litter effect did not explain a substantial proportion of the phenotypic variance for any hormone trait and was eliminated from the bivariate models to enhance convergence. The *P*-value for ${\hat{r}}_{g}\;$ being different from zero or from (negative) one was determined using a likelihood ratio test for bivariate models with and without the genetic correlation between traits set to 0 or (-1) 1, as described by Kayondo et al. (2025). Given the relatively small sample sizes for genetic parameter estimation and the resulting high standard errors associated with ${\hat{r}}_{g}$, combined with the novelty and relevance of the parameters estimated, significance of a test of ${\hat{r}}_{g}\;$ was declared at *P* ≤ 0.05, while those with *P* ≤ 0.25 were declared suggestively significant.

#### Genome-wide association studies

Results from univariate and bivariate GWAS analyses for the qNur hormone and backtest traits were reported by Kayondo et al. (2025). The same methods were used to conduct GWAS analyses for the cNur hormone traits, using the BayesB method implemented in the JWAS package (Cheng et al. 2018), with a chain length of 80,000, discarding the first 10,000 iterations as burn-in. Convergence of models was evaluated by visual inspection of trace plots of the sampled parameters. A QTL was defined as a nonoverlapping 1 Mb window that explained more than 1% of the estimated genetic variance (EGV) for the trait, computed as described by Wolc et al. (2012). A SNP was considered a lead SNP for a QTL if it had a posterior inclusion probability (PIP) >1%, which is the proportion of iterations of the Monte Carlo Markov Chain in which the SNP was included in the model.

Using bivariate analyses, pleiotropic QTL were detected based on the absolute difference (AD) in the posterior probability that a 0.25 Mb window had a positive vs a negative genetic covariance for the pair of traits, as described by Kayondo et al. (2025). Here, a 0.25 Mb window was considered a pleiotropic QTL if its AD value was greater than an arbitrary threshold of 0.02. We also assessed the effect of adjusting cNur stress hormone levels for qNur hormone levels, considering the hormone levels in the absence of major disease (i.e. hormone levels under NIS) as “baseline levels”. The log-transformed qNur level was added as a covariate in the univariate analysis of the log-transformed cNur level of the hormone. The resulting regression coefficient was then used to adjust cNur levels of each hormone for their qNur levels prior to their analysis in univariate GWAS.

Fine mapping of the identified QTL with major effects was performed following procedures described by Kayondo et al. (2025), involving imputation of genotypes in the 1 Mb QTL window to whole-genome sequence (WGS) using the SWine IMputation (SWIM) server (Ding et al. 2023), with the 650 K SNP panel as a background. A GWAS was then repeated using the imputed WGS genotypes in the 1 Mb window.

#### Candidate gene and functional enrichment analyses

Candidate genes for identified QTL were searched within a span of 5 Mb centered around the QTL using BioMart (Smedley et al. 2009) and the Ensembl pig gene database (Sscrofa11.1). The 5 Mb window was based on Pocrnic et al. (2024), who estimated it to capture 80% of the genetic variance in commercial pig populations.

**Fig. 1.**
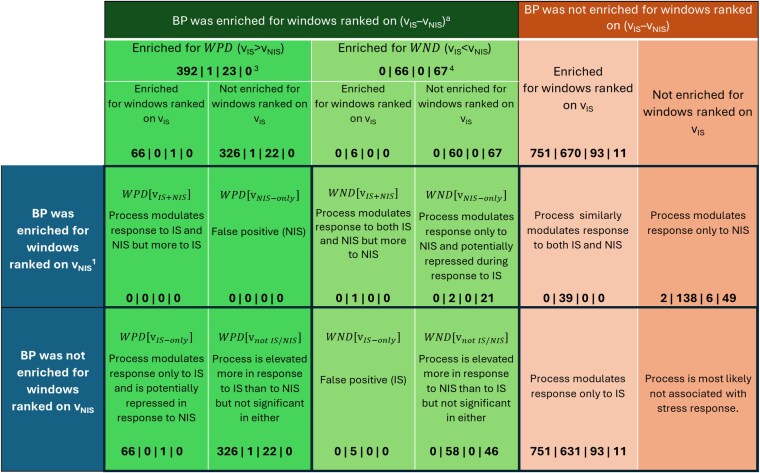
Postulates that define different classifications of biological processes (BPs) that are enriched for 0.25 Mb windows levels of a given stress hormone in hair grown under noninfectious stressors (NIS) and under infectious stressors (IS). The number of BPs in each category is the value below each postulate for the different hormones in the order: CL|CN|DH|DS. v_IS_ = %EGV explained by a 0.25 Mb window during response to IS; v_NIS_ = %EGV explained by a 0.25 Mb window during response to NIS; “v_IS_ > v_NIS_” indicates windows with a positive difference (WPDs) that explained higher %EGV under IS than NIS; “v_IS_ < v_NIS_” indicates windows with a negative difference (WNDs) that explained lower %EGV under IS than NIS; CL = cortisol, CN = cortisone, DH = DHEA, DS = DHEA-S. The difference in %EGV used to rank windows was v_IS_–v_NIS_. ^a^ v_IS_–v_NIS_ indicates the difference in %EGV explained by a window during IS and NIS. ^1,2^ For windows associated with hormone levels under NIS (from Kayondo et al. 2025) and under IS, respectively. ^3^ Total number of BPs enriched in ranked windows, respectively, associated with CL|CN|DH|DS that explained higher %EGV explained under IS than NIS. ^4^ Total number of BPs enriched in ranked windows, respectively, associated with CL|CN|DH|DS that explained lower %EGV explained under IS than NIS.

The limited power of GWAS for studies of this size results in many false negative results. Although it is not possible to differentiate false from true negative genomic regions, jointly, the genes that are located in nonsignificant genomic windows that explain a larger amount of variation for the trait than other regions can be used to explore and characterize biological processes (BPs) that are associated with the analyzed trait. For this purpose, ranked gene set enrichment analyses were performed with the gene set enrichment analysis (GSEA) software (Subramanian et al. 2005) using GWAS summary statistics, as described by Kayondo et al. (2025). Windows for the significant QTL that were identified by GWAS (>1% of EGV) were excluded from this analysis, to avoid enrichment results being driven by the significant QTL. Additionally, enriched BPs in 0.25 Mb windows that explained different percentages of the EGV for a given hormone during response to NIS (*v_IS_* = %EGV in hair collected at the end of qNur) vs during response to IS (*v_IS_* = %EGV in hair collected after exposure to disease in cNur) were evaluated using the difference *v_IS_-v_NIS_*. For this, a novel comparative gene set enrichment analysis (cGSEA) was used in which the 0.25 Mb windows were ranked based on this difference (*v_IS_–v_NIS_*), such that windows with a positive difference (*WPD*) were those that had greater %EGV during IS than NIS (i.e. *WPD(v_IS_ > v_NIS_)*), while windows with a negative difference (*WND*) were those that had lower %EGV during IS than NIS (i.e. *WND(v_IS_ < v_NIS_)*). BPs that were enriched in the *WPD* and *WND* for each hormone were then obtained using GSEA based on the library of GO terms constructed from the GO database (Subramanian et al. 2005). To differentiate GO terms that were enriched among *WPD* vs *WND*, the resulting q-values of enrichment, as computed by the GSEA software, were given positive vs negative signs. BPs enriched at FDR ≤ 0.25 were declared significant. To contrast results from the cGSEA with those from the ranked GSEA that was conducted separately for hair grown under NIS and IS, postulates in Fig. 1 were developed to guide the interpretation of the relevance of the BPs. To this end, a BP was categorized based on whether it was enriched in *WPD* or *WND* in the cGSEA analyses and based on whether it was significantly enriched in the ranked GSEA analyses for both IS and NIS [*v_IS_  _+_  _NIS_*], for only IS [*v_IS-only_*], for only NIS [*v_NIS-only_*], or for neither IS nor NIS [*v_not IS/NIS_*].

## Results and discussion

The experimental design of the natural disease challenge model used to generate these data was described in Putz et al. (2019) and Cheng et al.(2020) and included a quarantine nursery (qNur) that was intended to mimic high-health nucleus and multiplier farms of commercial breeding companies, allowing for the expression of genetic variation in stress response to NIS, such as weaning, transportation, social mixing, and human handling, most of which are part of routine management of pigs in nucleus breeding programs. It also included a disease challenge nursery (cNur) that was designed to mimic a commercial farm with high disease pressure, allowing for the expression of genetic differences in response to IS. Therefore, levels of stress hormones measured in hair grown up to the end of qNur informed on responses of pigs to NIS (see Kayondo et al. 2025 for details), while hormone levels measured in hair regrown during the challenge informed on responses of pigs to IS.

### Summary statistics

Distributions of natural log-transformed values of the hormone levels and of their ratios are in Supplementary Fig. 2. Table 1 shows summary statistics of hormone levels before log-transformation. Generally, average hormone levels were lower in hair grown during response to IS (in cNur) than the levels in hair of young pigs that were responding to NIS (in qNur) reported by Kayondo et al. (2025). Differences in average hormone levels in hair grown in qNur and cNur can reflect differences in the physiological response of pigs to NIS vs IS but could also be related to the difference in age of the pigs when these two hair samples were collected since age-related declines in levels of stress hormone levels have been observed in saliva of pigs (Ekkel et al. 1996; Ruis et al. 1997) and have also been observed in in humans (Pataky et al. 2021).

**Table 1.** Summary statistics for the concentrations of stress hormones (pg/mg) in hair regrown when exposed to disease, in addition to estimates of the effects of storing samples in ground form (with associated *P*-values), the proportion of phenotypic variance explained by litter effects, and of the phenotypic variance, with corresponding standard errors (SEs).

	No. of pigs	Mean	SD	Median	Min	Max
**Hormone levels (pg/mg)**						
** CL**	608	9.7	4.4	9.0	0.5	38.5
** CN**	478	9.2	5.2	8.2	0.1	36.4
** DH**	610	17.7	21.2	13.7	1.6	405.6
** DS**	339	4.4	8.1	1.8	0.0	64.1
** CL + CN (SOG)^a^**	471	18.5	7.8	17.1	1.9	56.5
** DH + DS (SOD)^b^**	331	22.0	12.1	19.4	2.2	64.4
**Hormone ratios**						
** CL/DH**	605	0.8	0.7	0.6	0.0	6.9
** CL/CN**	471	1.3	2.3	1.1	0.2	40.5
** CN/DH**	470	0.8	0.7	0.6	0.2	5.2
** CL/DS**	332	43.4	251.4	5.2	0.3	3548.5
** CN/DS**	331	52.1	308.3	4.6	0.2	3741.6
** DH/DS**	331	43.7	200.0	10.6	0.3	2462.5
** SOG/SOD**	328	1.2	1.0	0.9	0.2	10.5
** SOG/DH**	470	1.6	1.2	1.3	0.2	11.6

“na” indicates a parameter was dropped out of the model because the variance it explained was not significant.

CL = cortisol, CN = cortisone, DH = DHEA, DS = DHEA-S; SOGs = sum of glucocorticoids, SOD = sum of DHEA(S).

^a^Sum of glucocorticoids.

^b^Sum of DHEA(S).

In cNur hair, the average of the sum of DHEA(S) (DOS) was higher than the SOGs, which is consistent with the results reported by Kayondo et al. (2025) for the qNur hair samples. However, the average levels of CL (active form) and CN (inactive form) were relatively similar in cNur (9.7 vs 9.2 pg/mg), in contrast to levels in response to NIS, for which the level of CN was higher than that of CL (22.4 vs 15.8 pg/mg) (Kayondo et al. 2025). These results suggest a greater balance between the inactive and active forms of the glucocorticoids during IS, potentially aimed at achieving optimal glucocorticoid activities in response to physiological stress while avoiding the negative anti-inflammatory and catabolic effects from excessive exposure of the body to high cortisol. Additionally, in response to IS, the level of the active anti-glucocorticoid DH was on average higher than that of its inactive form DS (17.7 vs 4.4 pg/mg) and it was the most abundant hormone in hair grown in response to IS. Kayondo et al. (2025) reported substantially higher levels of DS than of DH in hair grown during response to NIS (257.6 vs 11.1 pg/mg). This could indicate that the activities of glucocorticoids are not only under regulation during IS by balancing CL and CN but also via the conversion of DS to DH.

### Heritability of stress hormone levels

**Fig. 2.**
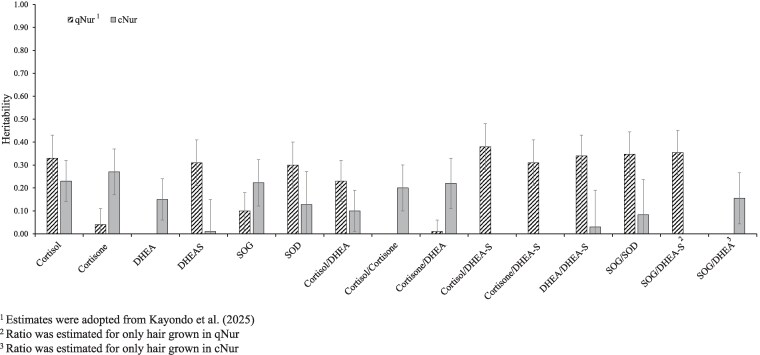
Heritability estimates (and standard error bars) for the natural log of stress hormone levels and of their ratios in hair grown during the quarantine nursery (qNur) and in hair regrown during the challenge nursery (cNur) under a natural polymicrobial challenge. SOG = cortisol + cortisone, SOD = DHEA + DHEA-S.

Estimates of heritability (${\hat{h}}^{2}$) for cNur stress hormone levels and their ratios are in Fig. 2, along with heritability estimates of the hormone traits in hair collected in qNur, as previously reported by Kayondo et al. (2025). The ${\hat{h}}^{2}$ for cNur hormone levels ranged from 0.01 to 0.27, with the ${\hat{h}}^{2}$ for CL being fairly similar to that for CN (0.27 ± 0.10 and 0.23 ± 0.09), which were both higher than the ${\hat{h}}^{2}$ for DH and DS (0.15 ± 0.09 and 0.01 ± 0.14). These differences between ${\hat{h}}^{2}$ are opposite to ${\hat{h}}^{2}$ reported by Kayondo et al. (2025) for hair collected on young healthy pigs, reflecting that genetics contributes more to the level of CN and DH in hair grown during response to IS than to NIS, and even less to the level of DS. However, these differences in ${\hat{h}}^{2}$ could also be due to the difference in age of the pigs. CL had a consistently moderate ${\hat{h}}^{2}$ under both NIS and IS (0.33 and 0.23, respectively).

The ${\hat{h}}^{2}$ for hormone ratios in cNur ranged from 0 to 0.22 (Fig. 2), with CN/DH having the highest estimate (0.22 ± 0.11). The ${\hat{h}}^{2}$ of ratios involving DS in hair grown under IS were estimated to be zero, because of its high phenotypic variance and low ${\hat{h}}^{2}$ compared to the other hormones. In contrast, these ratios were reported to have some of the highest ${\hat{h}}^{2}$ under NIS by Kayondo et al. (2025). Additionally, the ratio SOG/SOD had a much lower ${\hat{h}}^{2}$ in hair grown under IS compared to that reported by Kayondo et al. (2025) in hair grown under NIS (0.08 vs 0.35). Since the ${\hat{h}}^{2}$ of DS was near zero under IS, we also evaluated the ratio SOG/DH and found its ${\hat{h}}^{2}$ to be twice that of SOG/SOD (0.15 ± 0.11).

### Litter effects

Litter effects on levels of hormones in hair grown during response to IS were essentially absent (data not shown), while Kayondo et al. (2025) showed substantial litter effects in hair of young healthy pigs, ranging from 9% to 16% of phenotypic variance for hormone levels and from 0% to 15% for hormone ratios. The extent to which offspring perceive stressful events during their early life has been shown to be greatly influenced by maternal care in humans (Kazakou et al. 2023) and is also shaped by experiences in the early life environments in pigs (see review by Lucas et al. 2024). Unlike studies that have shown persistent effects of the maternal environment on the HPA axis in mice (Parfitt et al. 2004; Macrì and Würbel 2007) and in pigs (Miguel-Pacheco and Seddon 2021), our results suggest that litter effects did not progress to the older ages of these pigs.

### Genetic and phenotypic correlations

For reference, the full table with estimates of genetic parameters is included in Supplementary Table 1. In the following, specific estimates are highlighted and illustrated in corresponding figures and tables.

#### Among stress hormone traits under challenge

Table 2 summarizes estimates of genetic (${\hat{r}}_{g}$) and phenotypic (${\hat{r}}_{p}$) correlations among the hormone levels and their ratios under challenge. The ${\hat{r}}_{p}$ were substantial and positive between CL and CN (0.57 ± 0.03) and between DH and DS (0.60 ± 0.04), while estimates ranged from 0.11 to 0.28 for glucocorticoid levels (CL and CN) with DHEA(S). Positive ${\hat{r}}_{p}$ for hormone levels in hair grown under NIS were also reported by Kayondo et al. (2025). These positive ${\hat{r}}_{p}$ could be related to the leader–follower functional mode of operation of these hormones during stress response and align with the known increase in levels of glucocorticoids above normal circadian levels in response to stress, followed by homeostatic antiglucocorticoid actions of DH and DS (Kalimi et al. 1984; McNelis et al. 2013; Son et al. 2018).

**Table 2.** Estimates (standard errors) of genetic (below diagonal) and phenotypic (above diagonal) correlations among log-transformed stress hormone levels in hair regrown when exposed to disease.

	CL	CN	DH	DS	CL/DH	CL/CN	CN/DH	CL/DS	CN/DS	DH/DS
**CL**		0.57 (0.03)	0.28 (0.04)	0.21 (0.06)	0.51 (0.03)	0.17 (0.05)	0.28 (0.05)	0.10 (0.06)	−0.01 (0.06)	−0.17 (0.06)
**CN**	0.35 (0.30)^c^		0.25 (0.05)	0.11 (0.06)	0.23 (0.05)	−0.76 (0.02)	0.75 (0.02)	0.06 (0.06)	0.35 (0.05)	−0.06 (0.06)
**DH**	0.35 (0.33)	0.59 (0.56)		0.60 (0.04)	−0.71 (0.02)	−0.03 (0.05)	−0.54 (0.04)	−0.62 (0.04)	−0.61 (0.04)	−0.38 (0.05)
**DS**	0.61 (0.49)^a^	−0.09 (0.60)	0.85 (0.41)^d^		−0.34 (0.05)	0.09 (0.06)	−0.29 (0.06)	−0.96 (0.00)	−0.92 (0.01)	−0.96 (0.00)
**CL/DH**	0.61 (0.22)^a^	0.15 (0.35)	−0.53 (0.28)	0.08 (0.66)		0.18 (0.15)	0.69 (0.03)	0.55 (0.04)	0.46 (0.04)	0.13 (0.06)
**CL/CN**	0.26 (0.28)	−0.73 (0.18)^a^	−0.20 (0.41)	0.46 (0.51)	0.37 (0.32)^b^		−0.66 (0.03)	−0.01 (0.06)	−0.40 (0.05)	−0.07 (0.06)
**CN/DH**	0.35 (0.32)	0.92 (0.12)^a^	−0.15 (0.43)	−0.39 (0.54)	0.49 (0.28)	−0.82 (0.17)^a^		0.39 (0.05)	0.60 (0.04)	0.12 (0.06)
**CL/DS**	0.24 (0.55)	0.35 (0.54)	−0.56 (2.54)	−0.89 (0.17)	0.51 (0.63)	−0.38 (0.56)	0.68 (0.58)		0.94 (0.01)	0.93 (0.01)
**CN/DS**	0.24 (0.45)	nc	nc	−0.91 (0.18)^a^	0.08 (0.60)	−0.80 (0.47)^a^	0.87 (0.33)^b^	0.88 (0.24)		0.89 (0.01)
**DH/DS**	−0.49 (1.63)	−0.18 (0.75)	0.02 (1.70)	−0.94 (0.10)^a^	−0.33 (0.82)	−0.33 (0.63)	0.23 (0.59)	0.79 (0.34)	0.71 (0.51)	

nc = Did not converge; estimate is significantly different from zero: ^a^ = *P* ≤ 0.05, ^b^ = (*P* ≤ 0.15), ^c^ = (*P* ≤ 0.25).

^d^ = Estimate is not significantly different from one at *P* ≤ 0.05.

CL = cortisol, CN = cortisone, DH = DHEA, DS = DHEA-S, SOGs = sum of glucocorticoids, SOD = sum of DHEA(S).

The ${\hat{r}}_{p}$ of hormone levels with hormone ratios ranged from low to very high, with ratios with DS in the denominator having the highest negative ${\hat{r}}_{p}$ with DS (−0.92 to −0.96) because of the large phenotypic variance of DS compared to the other hormones (Table 2). Similarly, ${\hat{r}}_{p}$ among hormone ratios ranged from very low (−0.01 between CL/DS and CL/CN) to very high (0.94 between CN/DS and CL/DS).

Genetic correlation estimates were moderate and positive between all hormone levels (0.35 to 0.85) (Table 2), except for an essentially zero estimate between CN and DS. The ${\hat{r}}_{g}$ between CL and CN was lower in hair grown under IS compared to that reported by Kayondo et al. (2025) for hair collected in qNur (0.99 vs 0.35). This comparison could not be made for the DHEA(S) because DH under NIS was not heritable. However, the ${\hat{r}}_{g}$ between DH and DS in cNur was high (0.85 ± 0.41) and not significantly different from one (*P* ≤ 0.05), suggesting they had a similar genetic basis under the disease challenge.

For the ${\hat{r}}_{g}$ between hormone levels and hormone ratios under challenge, CL had a moderately high positive ${\hat{r}}_{g}$ with CL/DH (Table 2), while CN had a high negative ${\hat{r}}_{g}$ with CL/CN and a high positive ${\hat{r}}_{g}$ with CN/DH. As expected, DS had a strong negative ${\hat{r}}_{g}$ with all ratios that had DS as the denominator (−0.89 to −0.94). The ${\hat{r}}_{g}$ between the hormone ratios ranged from low to high (−0.87 to 0.82), with CL/CN having a positive ${\hat{r}}_{g}$ with both CL/DH and DH/CN but a negative ${\hat{r}}_{g}$ with CN/DS. As expected, based on the arrangement of hormones in the respective ratios, CN/DS had a high positive ${\hat{r}}_{g}$ with CN/DH.

#### Between hormone traits in qNur and cNur hair samples

**Fig. 3.**
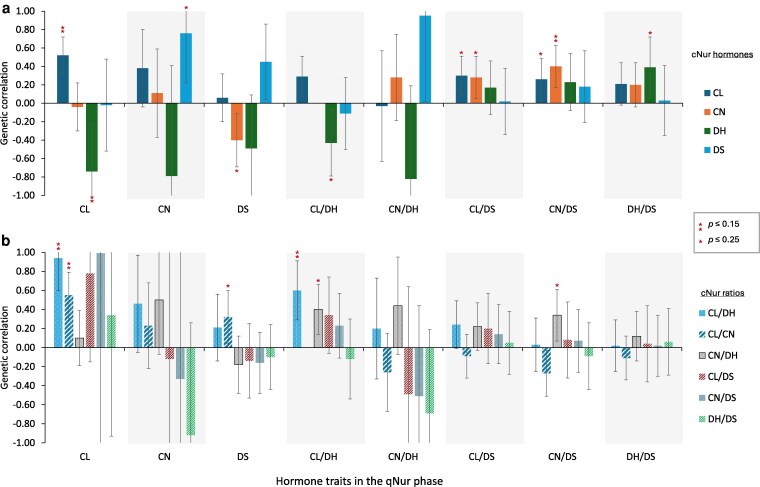
Estimates (SE) of genetic correlations (and standard error bars) between a) log-transformed hormone levels and b) between hormone ratios in hair grown during the challenge nursery phase of the natural disease challenge model with hormone traits in the quarantine nursery phase. CL = cortisol, CN = cortisone, DH = DHEA, and DS = DHEA-S.

Estimates of ${\hat{r}}_{p}$ between qNur and cNur hormone traits are in Supplementary Fig. 3 and ranged from −0.16 to 0.30. Figure 3 shows the corresponding ${\hat{r}}_{g}$. Bivariate analyses involving DH and CL/CN under NIS did not converge because of their very low ${\hat{h}}^{2}\;$(see Kayondo et al. 2025). The ${\hat{r}}_{g}$ were generally positive between levels of the same hormone grown under NIS and under IS but only the ${\hat{r}}_{g}$ for CL was significantly different from zero (*P* ≤ 0.15) at 0.52 ± 0.20. This ${\hat{r}}_{g}$ was also significantly different from one (*P* < 0.05), suggesting that CL in hair grown under NIS and under IS have some genetic similarity but are different traits. However, when genotype for the major QTL for CL that was identified for CL in qNur by Kayondo et al. (2025) was fitted as a covariate for both traits, the ${\hat{r}}_{g}$ between CL grown under NIS vs IS dropped from 0.52 ± 0.20 to essentially zero (0.13 ± 0.32), suggesting that this ${\hat{r}}_{g}$ is mostly driven by this major QTL.

CN under IS had a negative ${\hat{r}}_{g}$ with only DS under NIS, while DH under IS had a negative ${\hat{r}}_{g}$ with levels of CL, CN, and DS under NIS but only the ${\hat{r}}_{g}\;$ with CL approached significance (*P* < 0.15). Generally, the very low ${\hat{h}}^{2}\;$ for CN and DH under NIS relative to their moderate ${\hat{h}}^{2}$ under IS (0.04 vs 0.27 and 0 vs 0.15) suggests that some genes modulate CN and DH levels in hair grown under IS but not under NIS. Similarly, there is potentially a set of genes or gene elements that modulate the level of DS under NIS but that do not participate in its modulation in hair grown under IS. Overall, these results indicate that CL in hair of young healthy pigs has potential as a genetic indicator of CL in hair grown under IS, while levels of other hormones evaluated (i.e. CN, DH, and DS) in hair grown under NIS showed limited predictive potential for their respective levels in hair grown under IS.

Generally, the ${\hat{r}}_{g}$ of hormone ratios under NIS with those under IS did not reach significance at *P* ≤ 0.05 (Fig. 3) but some were suggestive at *P* ≤ 0.15. The ${\hat{r}}_{g}$ of hormone levels under NIS with hormone ratios under IS generally exhibited trends as expected based on the ${\hat{r}}_{g}$ between the respective hormones involved in the ratio. Among the ratio traits, CL/DH under IS had a positive ${\hat{r}}_{g}$ with CL under NIS, while CL/CN under IS had a positive ${\hat{r}}_{g}$ with both CL and DS under NIS. Only CL/DH and CN/DH under IS had a moderately high positive ${\hat{r}}_{g}$ with their respective ratios under NIS. CN/DH under IS also had a moderate positive ${\hat{r}}_{g}$ with CL/DH under NIS. Both CL/DH and CL/CN under IS had a moderate positive ${\hat{r}}_{g}$ with CL under NIS, while CL/CN under IS also had a moderate positive ${\hat{r}}_{g}$ with DS under NIS. However, although some ratios under NIS had a strong ${\hat{r}}_{g}$ with hormone levels under IS, the use of a ratio as a genetic predictor of levels of individual hormones under IS may not be relevant.

#### Between hormone levels in hair grown during the challenge and backtest responses

Estimates of ${\hat{r}}_{g}$ and ${\hat{r}}_{p}$ between hormone traits under NIS with backtest responses were reported by Kayondo et al. (2025). In this study, the relationships of backtest responses with hormone traits of hair grown under IS were evaluated. Estimates of phenotypic correlations for IS hormone traits with backtest responses were very low, ranging from −0.08 to 0.08 (Supplementary Table 2). Kayondo et al. (2025) also reported very low ${\hat{r}}_{p}$ of backtest responses with hormone traits in hair grown under NIS. These low ${\hat{r}}_{p}$ could be due to the nature of responses involved, with the backtest responses being more physical and behavioral and to a greater extent capturing response to acute handling stress, while stress hormone levels in hair measure accumulated responses to acute or chronic stressors faced over longer periods.

**Fig. 4.**
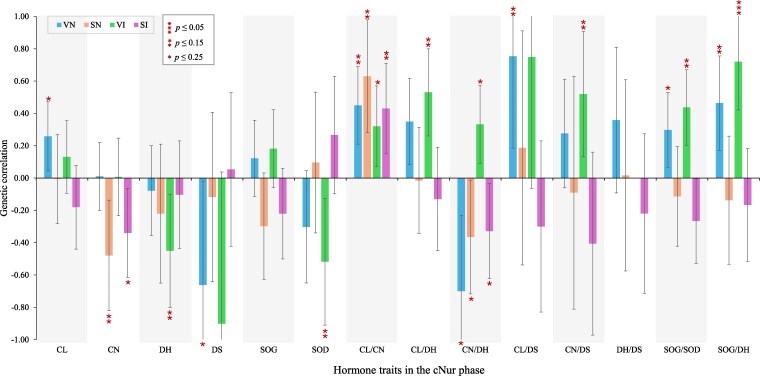
Genetic correlation estimates and standard error bars of responses to the 30 s backtest performed on young and healthy pigs at ∼27 d of age with natural log-transformed stress hormone levels in hair regrown during the challenge nursery phase of the natural disease challenge. Estimates that were significantly different from zero are shown by (***P ≤ 0.05, **P ≤ 0.15; *P ≤ 0.25). CL = cortisol, CN = cortisone, DH = DHEA, DS = DHEA-S, SOG = CL + CN, SOD = DH + DS, VN = vocalization number, VI = vocalization intensity, SN = struggles number, and SI = struggles intensity.

The ${\hat{r}}_{g}$ of hormone traits in hair grown under IS with backtest responses generally ranged from low to moderately high (Fig. 4) but none of the estimates were significantly different from zero (*P* > 0.05). Considering ${\hat{r}}_{g}$ that were suggestively significant (*P* ≤ 0.25), high backtest scores tended to be genetically correlated with lower hormone levels in hair grown under IS, except for CL, which had suggestively positive ${\hat{r}}_{g}$ with VN. Additionally, high scores for all backtest responses had suggestively positive ${\hat{r}}_{g}$ with CL/CN (0.32 to 0.63, *P* ≤ 0.15), while vocalization traits (VN and VI) had suggestively positive ${\hat{r}}_{g}$ with both CL/DH and CL/DS (*P* ≤ 0.15). However, more data are required to validate these genetic correlations. It should also be noted that the backtest responses are strongly genetically correlated with each other, in particular the number and intensity of the same response (struggling or vocalization), as reported by Kayondo et al. (2025).

The results in Fig. 4 also generally suggest that selecting pigs with high backtest scores may result in pigs with relatively lower HPA activity during response to IS, characterized by lower stress hormone levels in their hair. Studies that used backtest responses to categorize pigs as proactive (active) and reactive (passive) have also shown that active pigs tend to have lower HPA reactivity than the passive ones (Koolhaas et al. 1999, 2007). Therefore, responses to a backtest performed on young and healthy pigs have potential as a genetic indicator of their physiological stress response to IS. This relationship could be due to the coping style of an individual involving the recruitment of various neuro-physiological and physical mechanisms which, together, may enable an individual to survive in a challenging environment (Folkman and Moskowitz 2004). Additionally, while this study used castrated pigs, differences in backtest responses may be observed between castrated and uncastrated males, as well as between males and females. Interestingly, Kanitz et al. (2019) showed no significant sex differences in coping style between female and uncastrated male pigs.

### Genome-wide association studies

#### Univariate GWAS

**Fig. 5.**
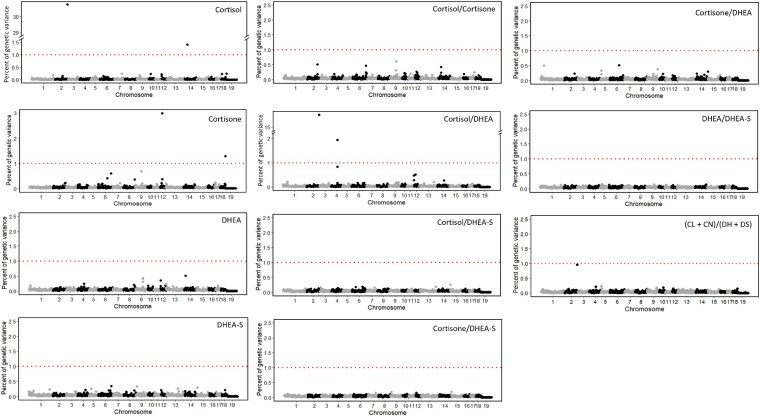
Manhattan plots for the percentage of genetic variance explained by nonoverlapping 1 Mb windows for the natural log-transformed stress hormone levels and their ratios in hair regrown during the challenge nursery phase of the natural disease challenge model. A window was considered a QTL if it explained more than 1% of the estimated genetic variance (above the threshold line). CL = cortisol, CN = cortisone, DH = DHEA, DS = DHEA-S.

GWAS results for stress hormone levels in hair grown prior to the disease challenge (under NIS) were reported by Kayondo et al. (2025). Results of the univariate GWAS for hormone levels and their ratios in hair grown during the disease challenge (under IS) are shown in Fig. 5. The QTL identified for different hormone traits in hair grown under IS are summarized in Table 3.

**Table 3.** QTL identified for the levels of stress hormones and their ratios in hair regrown when exposed to disease, the percentage of the estimated genetic variance they explained, lead SNPs with a posterior inclusion probability (PIP) ≥ 1%, and candidate genes identified in 5 Mb regions centered on the QTL window.

SSC	1 Mb window	Trait	% genetic variance	Lead SNP (PIP)	Candidate genes
	144	CL	37.8	rs81333622 (1.00)	*ARHGAP26, NR3C1, HDAC3, SRA1*
2		CL/DH	18.0	rs331270660 (0.30)	
4	72	CL/DH	1.9	rs341325631 (0.01)	*LOC106510078, RAB2A*
12	24	CN	3.0	rs319716381 (0.03)	*NR1D1, OSBPL7, ARHGAP23, SOCS7*
14	30	CL	1.4	rs343634627 (0.01)	*NCOR2, KDM2B, KMT5A*
19	5	CN	1.3	rs329868200 (0.05)	*ARHGAP6, STS, TBL1X*

SSC = *Sus scrofa* chromosome; CL = cortisol, CN = cortisone, DH = DHEA, DS = DHEA-S; PIP = posterior inclusion probability.

The univariate GWAS identified a major QTL at 144 Mb on SSC2 for both CL (37.8% of EGV) and CL/DH (18% of EGV) (Table 3). This major QTL was also identified by Kayondo et al. (2025) and explained 45.3% and 1.2% for CL and CL/DS in hair grown under NIS. Additional QTL with smaller effects were also identified, explaining from 1.3% to 3.0% of EGV for CL, CN, and CL/DH on SSC 4, 12, 14, and 19. No QTL was identified for DH or DS. Kayondo et al. (2025) also reported no QTL for DH in hair grown under NIS but did identify a QTL on SSC3 for DS and CL/DS and other QTL for CL/DS and CN/DS on SSC 5, 11, and 13. These QTL were not identified for hormone levels in hair grown under IS.

Regression coefficients (on the log scale) for CL, CN, DH, and DS adjusted for respective levels in hair grown under NIS (as baseline levels) were 0.30 (*P* < 0.001), 0.09 (*P* = 0.07), 0.05 (*P* = 0.31), and 0.07 (*P* = 0.24), respectively. Since the adjustment was only significant for CL, its effect on GWAS results was only evaluated for CL. Supplementary Fig. 4 shows the resulting Manhattan plot, indicating that the QTL on SSC2 dropped from 37.8% to 32.9% of EGV, while corresponding estimates for the QTL on SSC14 were 1.4% and 0.9%, suggesting no substantial effect of this adjustment. Based on these results, GWAS results presented in the following were based on unadjusted hormone levels.

Candidate genes for the major QTL for CL on SSC2 were the same as discussed for CL in hair grown under NIS by Kayondo et al. (2025), including the *NR3C1, SRA1*, and *HDAC3* genes, except with the addition of the *ARHGAP26* gene. The ARHGAP26 protein is a negative regulator of the Rho family that converts the small GTP-binding protein RhoA (GTP-RhoA) to its inactive GDP-bound form (Ohta et al. 2016; Chen et al. 2019). Candidate genes in the QTL region for CL on SSC14 included *NCOR2, KDM2B,* and *KMT5A* (Table 3). The nuclear receptor corepressor (*NCOR2*) modulates the activities of the GR by recruiting histone deacetylases (HDACs) to silence gene expression by altering chromatin structure (Bailey et al. 1999). Other candidate genes identified in the QTL regions on SSC4 for cNur CL/DH include an uncharacterized translation initiation factor IF-2-like (LOC106510078) and the *RAB2A* gene, which encodes a small GTPase that functions as a molecular switch, cycling between an active GTP-bound state and an inactive GDP-bound state.

Candidate genes in the QTL region on SSC12 for CN in hair grown under IS include *NR1D1, OSBPL7, ARHGAP23*, and *SOCS7*. The *NR1D1* (Nuclear Receptor Subfamily 1 Group D Member 1) gene product is part of the core molecular clock system (Yang et al. 2007) and could regulate circadian levels of CN by regulating the expression of the *BMAL1* gene. The *OSBPL7* gene encodes a member of the oxysterol-binding protein (OSBP) family with a highly conserved C-terminal OSBP-like sterol binding domain. OSBPs bind oxysterols, which function as intermediates in the synthesis of steroid hormones (Björkhem and Diczfalusy 2002; Olkkonen et al. 2012). A member of the OSBP family, *OSBPL11* was also reported as a candidate in a QTL region for the ratio of CN/DS in hair of pigs grown during response to NIS (Kayondo et al. 2025). The *ARHGAP23* gene encodes the Rho GTPase activating protein 23. The *SOCS7* gene product inhibits cytokine signaling by interacting with JAK kinases or cytokine receptors, terminating the signaling cascade (Cooney 2002). This could modulate inflammatory cytokines during stress, which activates the HPA axis and stimulates CL production.

Candidate genes in the QTL region on SSC19 for CN under IS included the *ARHGAP6, STS,* and *TBL1X* genes (Table 3). The *STS* gene encodes for the sulfotransferase (STS) enzyme, which catalyzes the conversion of inactive sulfated steroids, such as DHEA-S, into their active forms, such as DHEA. Its relationship with CN levels in hair is indirect but, during stressful conditions, DHEA can influence adrenal gland function and the production of glucocorticoids, including CL and CN. Also, by modulating the availability of the enzyme 11β-hydroxysteroid dehydrogenase (11β-HSD) type 1 (McNelis et al. 2013), STS can indirectly influence the levels of CN and CL in circulation. The *TBL1X* gene encodes a protein that is part of co-repressor complexes such as the NCoR/SMRT (Silencing Mediator of Retinoid and Thyroid Receptors) complex (Perissi et al. 2004; Pray et al. 2022) and thus could participate in transcriptional regulation of genes involved in inflammation and in steroid hormone metabolism.

#### Pleiotropic QTL for stress responses under NIS and IS

Bivariate GWAS was performed to identify nonoverlapping 0.25 Mb windows that were associated with pleiotropy for levels of a given hormone in hair grown under NIS vs IS (Supplementary Fig. 5), as well as for pairs of backtest responses and stress hormone levels in hair grown under IS (Supplementary Fig. 6). Windows associated with pleiotropy for pairs of backtest responses and hormone levels in hair grown under NIS were reported by Kayondo et al. (2025). A window was considered a potential pleiotropic QTL if it had an AD value ≥ 0.02. Based on this threshold, among all hormone traits evaluated, only the major QTL on SSC2 was identified as pleiotropic for CL in hair grown under NIS and IS (AD value = 1.0). As mentioned above, this major QTL drives most of the genetic correlation between CL under NIS and IS. For hormone levels and backtest responses, only one pleiotropic QTL was identified, on SSC12 for SN and CN (AD value = 0.02).

#### Fine mapping the major QTL on SSC2

**Fig. 6.**
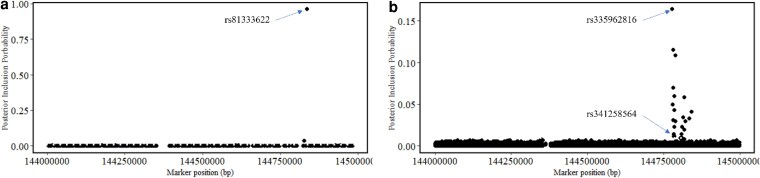
Scatter plots showing the posterior inclusion probability of SNPs in the QTL at 144 Mb on SSC2 for cortisol under challenge from the GWAS performed using a) the 650 K SNP panel and b) imputed SNPs in the 1-Mb QTL window at the whole-genome sequence density.

The GWAS performed using the imputed 650 K SNP panel identified SNP rs81333622 as the lead SNP for the QTL on SSC2 for CL in hair grown under IS, with PIP =1.0 (Fig. 6a). In hair grown under NIS, Kayondo et al. (2025) reported SNP rs336612332 as the 650 K lead SNP for this same QTL, with PIP = 1.0, and also showed that it was in very high linkage disequilibrium (LD) (r^2^ = 0.91) with the lead SNP (rs81333622) we found in this QTL for hair grown under IS. Interestingly, both these lead SNPs (rs81333622 and rs336612332) were found to be associated with plasma cortisol concentration measured 1 h after an ACTH injection of large white pigs by Terenina et al. (2025), which are denoted by AX-116190813 and AX-116190798, respectively. Fitting the genotype for this lead SNP (rs81333622) as a fixed covariate in the GWAS model for CL explained the full effect of the QTL for CL in hair grown under IS, suggesting that it was in very high or complete LD with the causative mutation for this QTL.

**Fig. 7.**
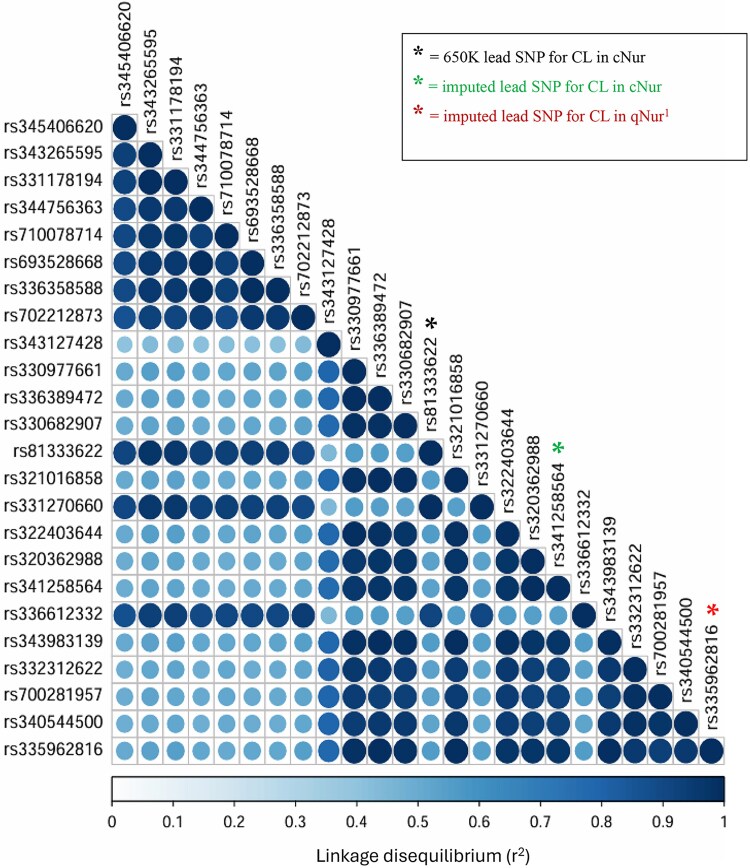
Linkage disequilibrium between the 21 SNPs identified with a posterior inclusion probability greater than or equal to 1% from the GWAS performed with SNPs in the QTL window for cortisol during the challenge phase at 144 Mb on SSC2 imputed to whole-genome sequence. Highlighted SNPs include the lead SNP that was identified for cortisol by the GWAS using the 650 K SNP panel (by *) and the imputed lead SNPs identified by the GWAS with SNPs in the major QTL imputed to whole-genome sequence for cortisol levels of hair grown under the disease challenge(by *) and in the quarantine nursery (by *) (^1^ as reported by Kayondo et al. (2025).

To get closer to the potential causal variant, the GWAS for CL was repeated with SNPs in this 1-Mb QTL window at 144 Mb on SSC2 imputed to WGS. In this GWAS, 21 imputed SNPs were identified to have an elevated PIP, ranging from 0.01 to 0.16 (Fig. 6b), including SNP rs341258564, which was reported by Kayondo et al. (2025) as the WGS-imputed lead SNP for CL in hair grown under NIS, with a PIP of 1.0. These SNPs were mostly located within 60 kb upstream of the 650 K lead SNP (rs81333622), while one was located 5 kb downstream. The SNP rs335962816 was the WGS-imputed lead SNP for CL under IS, with the highest PIP (0.16). The LD between the WGS-imputed lead SNP for CL in cNur, the 650 K lead SNP, and other SNPs in the QTL region with PIP > 1% are shown in Fig. 7. The WGS-imputed lead SNP (rs335962816) was in moderate LD (r^2^ = 0.56) with the 650 K lead SNP (rs81333622) but in very high LD (r^2^ = 0.97) with the WGS-imputed lead SNP for CL under NIS (rs341258564) reported by Kayondo et al. (2025). Fitting the genotype of the WGS-imputed lead SNP for CL under IS as a fixed covariate in the GWAS model reduced the %EGV of the major QTL for CL and CL/DH from 37.8% and 18.0%, respectively (Fig. 5) to about 2% for both. Similarly, fitting the genotype of the WGS-imputed lead SNP for CL under NIS (i.e. rs341258564) as a fixed covariate reduced the %EGV of this major QTL to 1.8% and 3.6% for, respectively, CL and CL/DH under IS. Additionally, fitting the genotype of the WGS-imputed lead SNP for CL under IS as a fixed covariate for CL under NIS dropped the %EGV for this QTL from 45.3% to 0.1%, showing that it captured the full effect of the major QTL for CL under NIS. Together, these results suggest that the WGS-imputed lead SNPs for CL under IS and NIS (rs335962816 and rs341258564, respectively) captured the effect of the same causal variant.

**Fig. 8.**
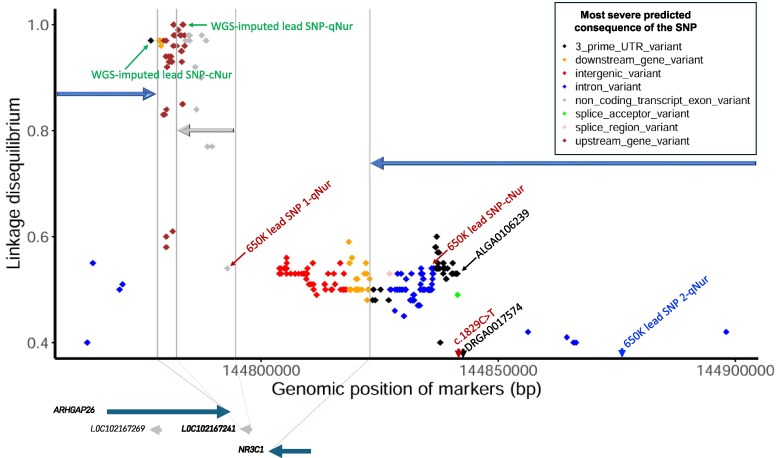
Predicted effects of SNPs in the QTL region for cortisol level that were in linkage disequilibrium (r^2^ ≥ 0.4) with the imputed lead SNP (rs335962816) on the genes in this region. The plot shows 650 K lead SNPs that were reported for cortisol levels in hair grown in qNur by Kayondo et al. (2025) and in cNur; the two WGS-imputed lead SNPs for cortisol in qNur and cNur; as well as the genomic position of the causative mutation (c.1829C > T) that was reported by Muráni et al. (2012) and two SNPs that were in very high LD with it (DRGA0017574 and ALGA0106239).

Based on the variant effect predictor (VEP) tool of Ensemble (McLaren et al. 2016), the WGS-imputed lead SNP (rs335962816) is most predicted to act as a 3′ UTR variant (Fig. 8). Since this SNP was in high LD with many other imputed SNPs, it could not be confirmed as the causative mutation. Within the same QTL region, Muráni et al. (2012), Reyer et al. (2016), and Terenina et al. (2025) showed that a mutation (c.1829C > T) in the *NR3C1* gene (rs335303636) was a major driver of CL levels in blood of pigs. Interestingly, Muráni et al. (2012) reported this causative mutation to be in high LD with SNP rs81333702 (or ALGA0106239), which was found to be in high LD with the 650 K lead SNP for CL under NIS (rs81333622) that was reported by Kayondo et al. (2025). For the pigs with data on CL under IS, however, the frequency of the minor allele at the causative mutation reported by Muráni et al. (2012), based on imputed genotypes, was 6% and its LD with the 650 K lead SNP and with the WGS-imputed lead SNPs for CL under IS was very low (less than r^2^ = 0.014) (Fig. 7).

Kayondo et al. (2025), using imputed genotypes, reported that the effect of the causative mutation identified by Muráni et al. (2012) (c.1829C > T) was essentially zero for CL in hair grown under NIS. Similarly, the estimate of the allele substitution effect of this imputed SNP on CL under IS was not significant (0.30 phenotypic standard deviations [PSD], *P* = 0.39) on the natural log scale, and it did not explain a substantial effect of the major QTL when fitted as a covariate in the GWAS model for CL under IS (data not shown), which was also observed for CL under NIS by Kayondo et al. (2025). Interestingly, when this causative mutation was simultaneously fitted as a fixed covariate in the model for CL under IS with the 650 K lead SNP (rs81333622) and the WGS-imputed lead SNP for CL under IS (rs335962816), the minor allele substitution effect estimates (in PSD) were highest for the WGS-imputed lead SNP (−0.59 ± 0.17, *P* < 0.001), followed by the 650 K lead SNP (−0.32 ± 0.15, *P* = 0.03) and then the causative mutation (0.23 ± 0.13, *P* = 0.07). Nevertheless, based on these results, and the fact that the causative mutation reported by Muráni et al. (2012) was imputed in our data, we cannot rule out the possibility that it also is the causative mutation for the major QTL we detected for CL in hair.

#### Effects of the major QTL for CL on other traits

Estimates for the allele substitution effects at our WGS-imputed lead SNP on natural log-transformed hormone traits evaluated in cNur are in Supplementary Table 3. Briefly, an extra copy of the minor allele (frequency = 9%) at the WGS-imputed lead SNP rs335962816 for CL in hair grown under IS was significantly associated (*P* < 0.001) with lower levels of CL on the original scale by 30 ± 4% and of CN under IS by 23 ± 6% but had no significant effect on levels of DH and DS under IS. Fitting separate covariates depending on breed of origin of the minor allele for the WGS-imputed lead SNP, derived as described by Kayondo et al. (2025), resulted in effect estimates that differed significantly between breeds (*P* < 0.05) of −1.09 ± 0.12 and −0.69 ± 0.15 PSD of CL under IS on the natural log scale for, respectively, Landrace and Yorkshire haplotypes. This analysis also indicated that the potential causative variant for this QTL that reduces cortisol was present at a low frequency (9%) in both Landrace and Yorkshire parental lines and potentially has a significantly larger effect for Landrace than for Yorkshire. Similar estimates were reported by Kayondo et al. (2025) for the breed-specific effects of the WGS-imputed lead SNP rs341258564 on CL under NIS but the difference in estimates between breeds was not significant at *P* < 0.05 in that study.

Of the 608 pigs with CL data under IS, 77.5% were homozygous for the major allele at the WGS-imputed lead SNP rs335962816, 0.8% were homozygous for the minor allele, and 21.7% were heterozygous. The WGS-imputed lead SNP for cNur CL was fixed for the major allele for one of the companies (details not shown) and there was a significant interaction (*P* < 0.001) between the SNP effect and company for CL and CN, but not for DH, DS, or any of the hormone ratios analyzed (Supplementary Table 3). Across companies, the estimated effect of an extra copy of the minor allele at this WGS-imputed lead SNP ranged from −0.36 to −1.63 PSD on the natural-log scale for CL and from −1.06 to 0.01 PSD for CN.

Adjusting for the lead SNP (rs335962816) by fitting it as a covariate for both traits in a bivariate model reduced the ${\hat{r}}_{g}$ between CL under NIS and IS from 0.52 ± 0.20 to essentially zero (0.13 ± 0.32), suggesting that this ${\hat{r}}_{g}$ was almost entirely driven by this major QTL. Additionally, when CL under IS was adjusted for CL under NIS as a baseline level, the WGS-imputed lead SNP still had a significant but relatively lower minor allele substitution effect on CL under IS (−0.26 ± 0.03 vs −0.36 ± 0.03 on the log scale), indicating that part of the effect of the QTL for CL under IS was independent of CL levels in hair grown during response to NIS and at a younger age. The ${\hat{r}}_{g}$ between CN under NIS vs IS was not different from zero (Fig. 3a) and was not affected by adjusting for the major QTL (0.11 vs 0.14). Similarly, adjusting for the WGS-imputed lead SNP (rs335962816) resulted in a nonsignificant (*P* = 0.88) increase in the ${\hat{r}}_{g}$ between CL and CN under IS from 0.35 to 0.40. Kayondo et al. (2025) also reported no effect of adjusting for their WGS-imputed SNP (rs341258564) on the ${\hat{r}}_{g}$ between CL and CN under NIS (${\hat{r}}_{g}$=0.99 before and after adjustment).

Adjusting for the major QTL also did not significantly affect the ${\hat{r}}_{g}$ of the level of CL under NIS with that of CN under IS or vice versa. Hence, there was no substantial evidence that the major QTL contributes differently to the ${\hat{r}}_{g}$ between CL and CN than the rest of the genome, under both NIS and IS.

#### Gene set enrichment analyses for responses to IS

**Fig. 9.**
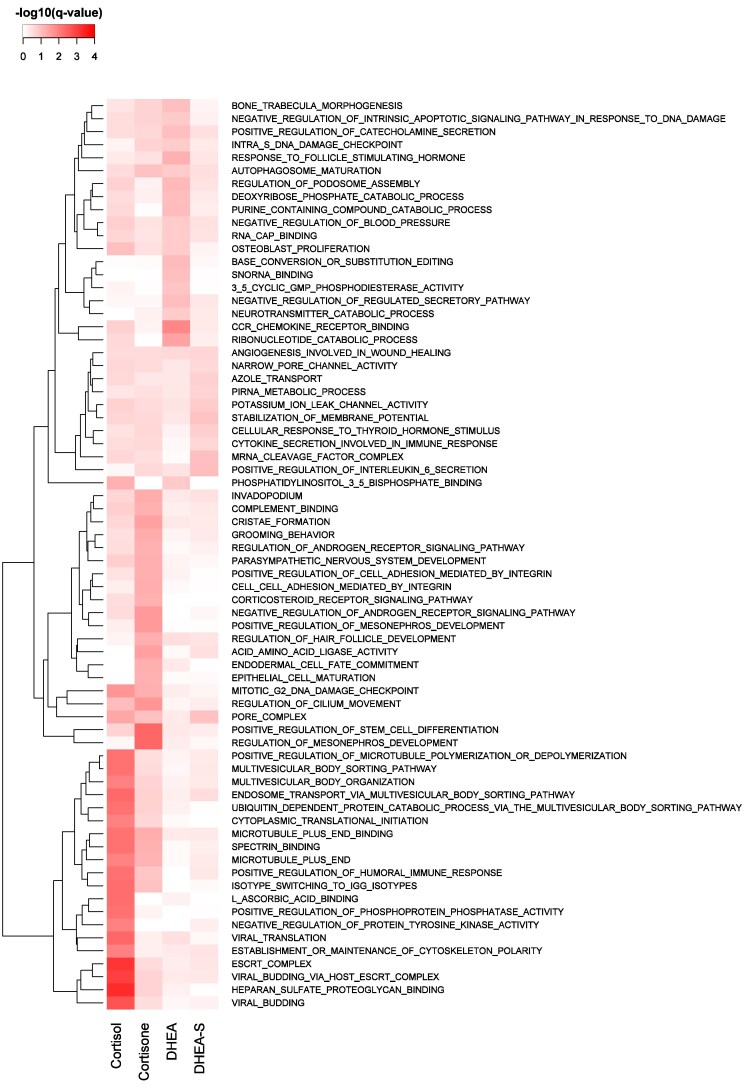
Heatmap with the top 20 most enriched (FDR ≤ 0.25) GO terms for at least one of cortisol, cortisone, DHEA, and DHEA-S in hair regrown during the challenge nursery phase of the natural disease challenge model.

Enrichment analyses for 0.25 Mb windows that were associated with stress hormone levels in hair grown during response to NIS were presented by Kayondo et al. (2025). For windows that were associated with stress hormone levels in hair grown under IS, a total of 817, 676, 94, and 11 BPs were significantly enriched (FDR ≤ 0.25) among the top-ranked windows beyond the identified QTL for CL, CN, DH, and DS, respectively (Supplementary Table 4). Of these, 200 BPs were significantly enriched for both CL and CN, including many BPs related to negative and positive regulation of immune-related processes, while no BPs were significantly enriched for both DH and DS. The heatmap in Fig. 9 shows the BPs that were among the top 20 most significantly enriched (FDR ≤ 0.25) BPs for at least one of the hormones analyzed. Windows associated with CL were strongly enriched (FDR ≤ 0.001) for BPs related to the viral-host cell interactions, viral translation, and multiplication. They were also enriched (FDR ≤ 0.25) for features related to enhancing humoral immune response, which involves the production of antibodies by B cells, primarily directed against extracellular pathogens such as bacteria, viruses, and toxins, as well as activating leukocytes. These immune responses also involve enhancing NK cell differentiation, boosting cell defense against fungus, and circulation of immunoglobulins and cytokines (especially IL-1, IL-2, IL-6, and IL-8). Also enriched in these windows were BPs related to bone marrow development coupled with the activity of B cells, as well as activation, differentiation, migration, and fusion of the different groups of leukocytes (Supplementary Table 4).

Windows that were associated with CN in hair grown under IS were enriched for BPs related to steroid dehydrogenase activities (Fig. 9), such as converting cortisone to and from cortisol. These windows were also enriched for processes related to immune cell activation and chemotaxis, as well as immunoglobulin production, which are processes involved in innate response to infection (Janeway et al. 2001), as well as for BPs that negatively regulate corticosteroid receptor signaling and cellular response to cholesterol and sterol. Some of the BPs that were significantly enriched for both CL and CN related to negative and positive regulation of immune-related processes and for BPs related to energy production, affirming the involvement of glucocorticoids in energy modulation during response to stress.

No BPs were significantly enriched for both DH and DS under IS. However, windows that were associated with DH were enriched (FDR ≤ 0.01) for BPs related to CC chemokine receptor binding (Fig. 9), which are key for viral entry into cells (Alkhatib 2009) and mediate migration of immune cells to sites of infection, tissue damage, or inflammation (Janeway et al. 2001 ). Other enriched BPs among windows associated with DH related to organ or tissue-specific immune response and respiratory burst, which is an immunological defense involving macrophages and neutrophils (Iles and Forman 2002). These windows were also enriched for features that regulate the cyclic AMP signaling pathway and its associated protein kinase activity, and for features that negatively regulate microtubule depolarization.

#### Comparative gene set enrichment analyses

**Fig. 10.**
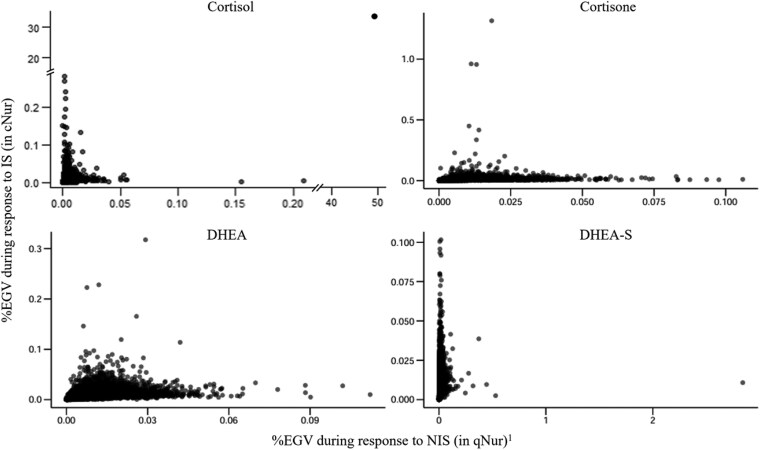
Percentage of the genetic variance (%EGV) explained by 0.25 Mb windows for levels of different hormones in hair when pigs were responding to noninfectious stressors (NIS, in qNur) and to infectious stressors (IS, in cNur). ^1^*Adopted from* Kayondo et al. (2025).

Enrichment analyses in the previous paragraph based on the %EGV explained by genomic windows showed that genomic regions that individually explained less than 1% of EGV can provide information on the BPs that modulate response to IS. However, comparison of GWAS results for the level of a given hormone in hair grown during response to IS (in cNur) vs NIS (i.e. prior to their entry in cNur) based on Kayondo et al. (2025) showed that 0.25 Mb windows can explain different %EGV for the same hormone in qNur vs cNur (Fig. 10). This is also reinforced by the ${\hat{r}}_{g}$ between respective hormone traits in qNur and cNur being less than 1 (Figs. 3a and 4b). Windows that differ in %EGV explained under NIS vs IS could harbor biological features that modulate response to either IS or NIS or to both at varying degrees. To investigate this, we proposed comparative GSEA (cGSEA) as a novel approach. Its main assumption is that the difference in %EGV explained by a 0.25 Mb window for levels of a given hormone during response to IS vs NIS is mostly attributed to the genetic control of a pig's physiological response to the level and/or type of stressors in qNur vs cNur, after accounting for other systematic effects in the GWAS models.

**Fig. 11.**
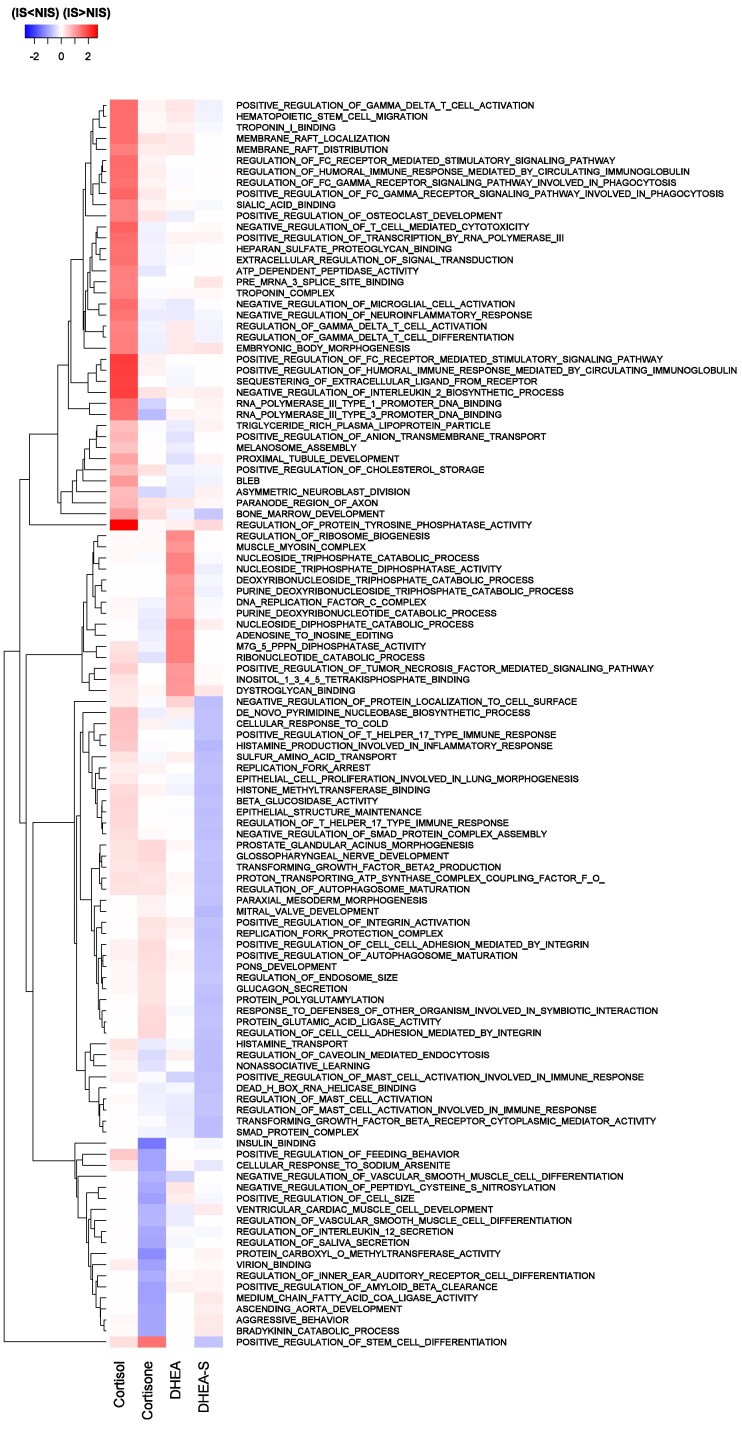
Heatmap showing the log10(q-value) of the top 30 GO terms that were significantly enriched (FDR ≤ 0.25) in windows that explained different %EGV for the levels of at least one of the hormones in hair between the qNur and cNur phases of the natural disease challenge model. WPDs = windows with a positive difference and WNDs = windows with a negative difference. A signed q-value was used such that a positive q-value indicated enrichment of the GO term among windows that explained more of the genetic variance for the hormone during the cNur than in the qNur phase.

Figure 11 summarizes the top 30 BPs that were enriched for at least one hormone among ranked *WPD* and *WND*, i.e. 0.25 Mb windows for which %EGV was significantly greater or smaller, respectively, under IS vs NIS. A detailed list of the BPs is in Supplementary Table 5. Figure 1 shows the number of BPs that were significantly enriched among 0.25 Mb windows associated with each hormone, categorized based on whether they were significantly enriched in windows ranked based on the %EGV during response to NIS (results adopted from Kayondo et al. 2025), during response to IS (see Supplementary Table 4), or based on the difference in %EGV in response to IS vs NIS.

The cGSEA results showed that for the active forms of the hormones (CL and DH), most of the significant BPs (FDR ≤ 0.25) were enriched among windows that explained more %EGV during IS than NIS (i.e. *WPD*: 392 for CL and 23 for DH) (Fig. 1). In contrast, for the inactive forms of the hormones (CN and DS), most of the significant (FDR ≤ 0.25) BPs were enriched among windows that explained less %EGV during IS than NIS (i.e. *WND*: CN = 66 and DS = 67). BPs in categories $WND[v_{IS-only}]$ and $WND[v_{not\;IS/NIS}]$ were enriched among windows that were associated with the inactive forms of the hormones (CN and DS), while those categorized as $WPD[v_{IS-only}]$ and $WPD[v_{not\;IS/NIS}]$ were mostly enriched among windows associated with the active forms of the hormones (CL and DH) (Supplementary Table 5). Almost all BPs in $WPD[v_{IS-only}]$ (66 of 67) were enriched in windows that were associated with CL but not with the other hormones and they were related to immune system activities such as T-cell selection, humoral immune response, natural killer differentiation, antigen receptor signaling, and microtubule depolymerization. These immune response pathways are potentially affected by the anti-inflammatory activities of CL during stress response.

The BPs in $WPD[v_{not\;IS/NIS}]$ for CL in Fig. 1 are also worth noticing, as they were not significantly enriched in windows based on the conventional GSEA but were found to explain more %EGV during IS than NIS. These included BPs involved in the breakdown of fatty acids and biosynthesis and/or metabolism of the resulting ketone bodies (Supplementary Table 5), and BPs that regulate saliva production and feeding behavior of pigs, i.e. processes that provide energy to the body. This category also included BPs related to modulating animal's temperament and estrous cycle.

Similar to CL, no GO terms were significantly enriched in *WND* for DH (Fig. 1 and Supplementary Table 5). Most BPs in windows associated with DH were classified as $WPD[v_{not\;IS/NIS}]\;$ (22) and as significantly enriched during IS but not NIS (93), suggesting that these BPs most likely participate in response to IS.

BPs in windows categorized as $WND[v_{IS-only}]$ and $WND[v_{not\;IS/NIS}]$ in Fig. 1 explained a greater %EGV during response to NIS than to IS and were mostly associated with the inactive forms of the hormones (i.e. CN and DS) (Supplementary Table 4). For DS, category $WND[v_{IS-only}]$ consisted of BPs related to the response of animals in defense from others (a case of social mixing) and to nonassociative learning. Both BPs are key aspects in the modern swine industry, where pigs are intensively managed. Nonassociative learning has been studied in invertebrates (Byrne and Hawkins 2015) and vertebrates (Best et al. 2008; Kirchkamp et al. 2012; Baragli et al. 2015) and is one aspect that could enable pigs to habituate and socialize with other littermates and to other concurrent stressors of weaning and/or getting used to moving mechanical equipment and humans in barns while exhibiting a diminished response to these NIS.

For CN, most of the significantly enriched BPs (FDR ≤ 0.25) were categorized as $WND[v_{not\;IS/NIS}]$ and $WND[v_{NIS-only}]$, i.e. they explained more %EGV under NIS than under IS, while only one BP was significantly enriched among *WPD*, and categorized as $WPD[v_{not\;IS/NIS}]$; a BP related to regulating TGF-β activation (Fig. 1). Generally, BPs enriched in windows that were associated with CN were related to negative regulation of B cell receptor signaling, IL-2 biosynthesis, and processes that positively regulate B-cell apoptotic processes, glycolytic processes, and autophagosome maturation (Supplementary Table 5).

We also observed BPs that were significantly enriched under either IS or NIS, or both, but that were not significantly enriched (FDR > 0.25) based on the cGSEA, suggesting that they were enriched in windows whose genomic contribution to the levels of the different hormones in hair may not vary significantly in hair grown under NIS and IS (the right two columns in Fig. 1). Of these, 39 BPs likely modulated CN to a similar degree under both IS and NIS and included BPs related to negative regulation of the body's defense response to viruses (Supplementary Table 5). The other BPs had been reported to be significantly enriched for hormone levels during response to NIS by Kayondo et al. (2025) and may be primarily involved in modulating the levels of hormones under NIS but not under IS. These BPs were also more associated with the inactive hormones (138 for CN and 49 for DS) than with the active hormones (2 for CL and 6 for DH), including BPs involved in the glycolytic pathway such as the metabolism of glucose-6-phosphate and fructose-6-phosphate, among others (Supplementary Table 5).

Many BPs were not significantly enriched based on the cGSEA but were enriched based on the conventional GSEA for hormone levels in hair grown under IS but not under NIS (751, 631, 93, and 11 for CL, CN, DH, and DS, respectively) (Fig. 1), indicating that they may be primarily involved in modulating levels of the hormones during IS but not significantly more than during NIS. These included BPs that were enriched for at least 3 of the hormones evaluated, including those related to energy homeostasis, hepatocyte growth factor signaling, and Schwann cell development (Supplementary Table 5).

Overall, the cGSEA was able to reveal deeper and more concise interpretations of the BPs involved in response to NIS and IS that were not captured by conventional GSEA based on enrichment among windows associated with hormone levels during one phase (i.e. NIS or IS). cGSEA can, therefore, be recommended to uncover biology behind genomic regions that do not reach significance in GWAS, especially when analyzing phenotypes that are evaluated under contrasting conditions.

## Conclusions

This is the first study to investigate the genetic basis of stress hormone concentrations in hair of pigs exposed to IS. When combined with our previous findings for hair grown under NIS (Kayondo et al. 2025), the results reveal key insights into the genetic architecture of stress response based on the levels of cortisol, cortisone, DHEA, and DHEA-S in hair of pigs. Levels in hair grown under IS were generally more heritable than levels of DHEA or DHEA-S. Levels of CL were heritable under both IS and NIS, those for CN and DH were heritable only under IS, while those for DS were heritable only under NISA major QTL near the glucocorticoid receptor on SSC2 affects the level of CL in hair grown under both NIS and IS and is the primary driver of the genetic correlation between CL levels in hair grown under IS and NIS. The minor allele at this QTL was associated with reduced CL and CN under both NIS and IS, suggesting the potential of using this QTL to select for higher or lower glucocorticoid levels. The previously identified causal c.1829C > T variant for CL levels in blood did not have a substantial association with CL in hair grown under IS. However, validation of these results through direct genotyping is recommended for future studies, as the most significant SNPs and the causal c.1829C > T variant were imputed.

A novel enrichment approach, cGSEA, was successfully used to identify BPs that were enriched in 0.25 Mb windows that explained different %EGV for hormone levels during response to NIS vs IS. Several BPs that were enriched among windows that explained a greater %EGV under IS than NIS were enriched for the active hormones (CL and DH) and these were mostly related to inflammatory response and energy metabolism. In contrast, for the inactive hormones (CN and DS), enriched BPs related to social interaction and nonassociative learning, and these were enriched in windows that explained less %EGV under IS than under NIS. Overall, these findings provide new insights into the genetic regulation of stress response in pigs under different stress environments.

Taken together, the results of this study support the use of noninvasive, heritable biomarkers such as levels of cortisol and cortisone in hair, as well as backtest responses, as potential genetic tools for selecting pigs that are better adapted to IS and NIS. However, given the large SE of many of the estimates, additional studies should be conducted to validate these findings.

## Supplementary Material

jkag005_Supplementary_Data

## Data Availability

The data analyzed in this study were collected on animals that were provided by and are part of the commercial breeding programs of the 7 original member businesses of the PigGen Canada research consortium. The data and samples generated on these animals are thus confidential and protected as intellectual property or as trade secrets. As a result, the data analyzed in this study are not publicly available but are stored in a secure database at the University of Alberta. Data can, however, be made available on reasonable request, as detailed in the supplementary data access procedure file attached. Supplemental material is available at G3 online.
