# Supplementary material for: Genetics of retroactive measures of stress response in pigs before and after exposure to a disease challenge
Source: G3 (Bethesda). 2026 Jan 13;16(3):jkag005. doi: 10.1093/g3journal/jkag005 (PMC12958817; doi:10.1093/g3journal/jkag005)
Supplement: jkag005_Supplementary_Data [file jkag005_supplementary_data.zip › Supplemental_Figure_1_G3-2025-406427.pdf]

Supplemental Figure 1: Plots showing the relationship between the levels of stress hormones extracted from hair samples and the length of time the ground samples were stored before hormone extraction.

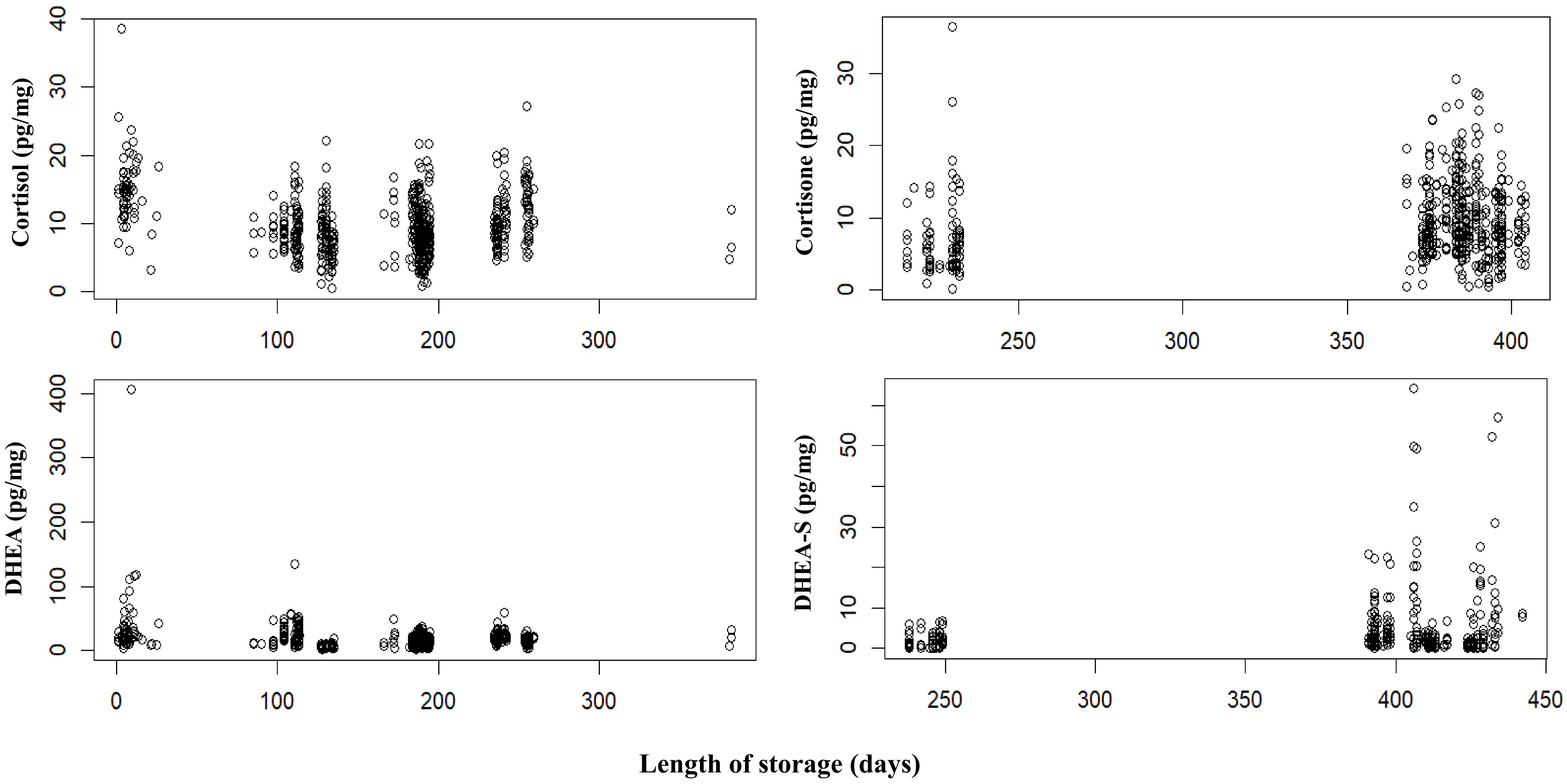
Cortisol (pg/mg)
Cortisone (pg/mg)
DHEA (pg/mg)
DHEA-S (pg/mg)
Length of storage (days)
