# Supplementary material for: Genetics of retroactive measures of stress response in pigs before and after exposure to a disease challenge
Source: G3 (Bethesda). 2026 Jan 13;16(3):jkag005. doi: 10.1093/g3journal/jkag005 (PMC12958817; doi:10.1093/g3journal/jkag005)
Supplement: jkag005_Supplementary_Data [file jkag005_supplementary_data.zip › Supplemental_Figure_2_G3-2025-406427.pdf]

Supplemental Figure 2: Distributions of natural log-transformed stress hormone concentrations (pg/mg) and their ratios in hair regrowth during the challenge nursery phase at ~82 days of age.

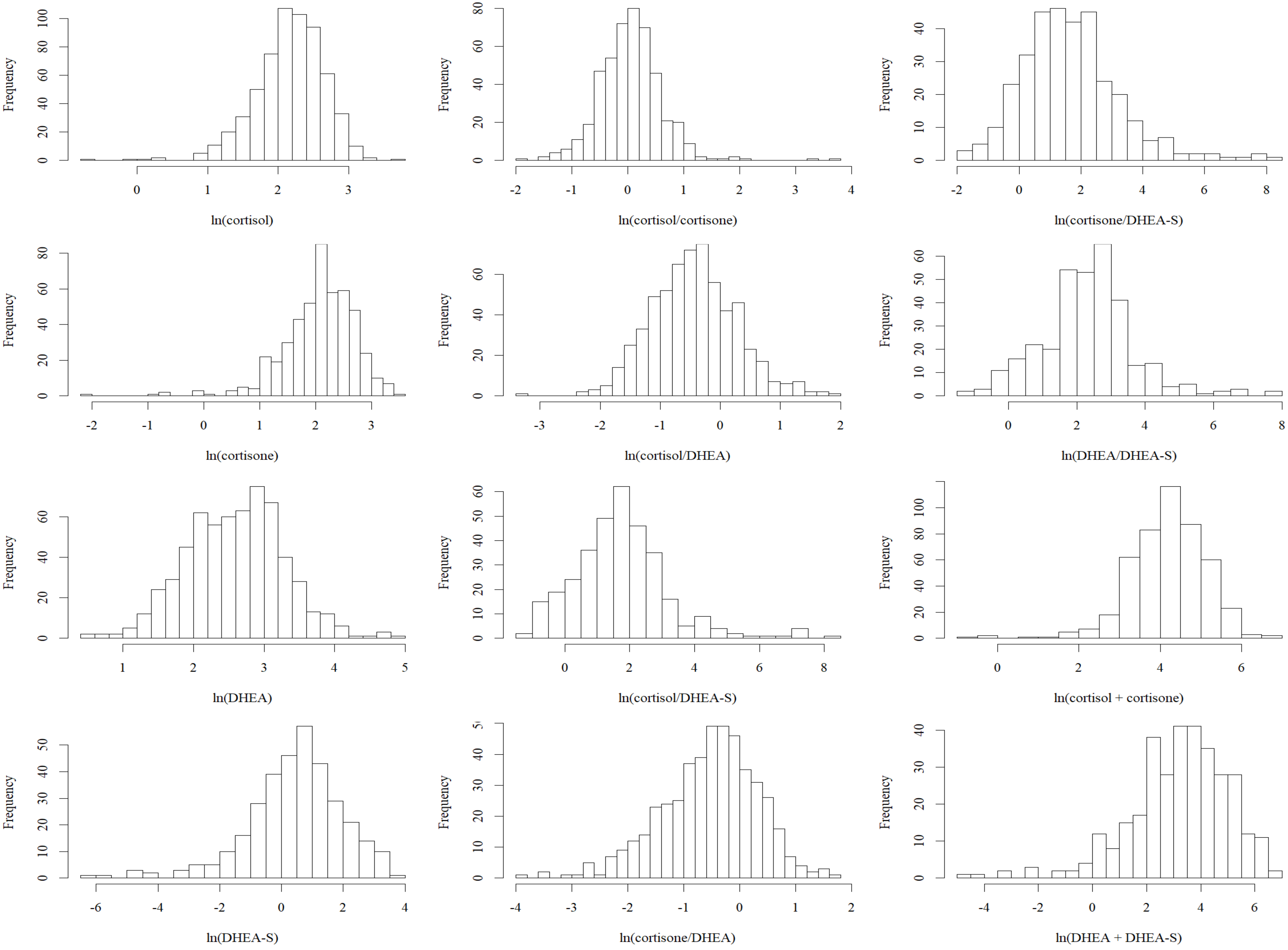
Frequency
ln(cortisol)
ln(cortisol/cortisone)
ln(cortisone/DHEA-S)
ln(cortisone)
ln(cortisol/DHEA)
ln(DHEA/DHEA-S)
ln(DHEA)
ln(cortisol/DHEA-S)
ln(cortisol + cortisone)
ln(DHEA-S)
ln(cortisone/DHEA)
ln(DHEA + DHEA-S)
