# Supplementary material for: Genetics of retroactive measures of stress response in pigs before and after exposure to a disease challenge
Source: G3 (Bethesda). 2026 Jan 13;16(3):jkag005. doi: 10.1093/g3journal/jkag005 (PMC12958817; doi:10.1093/g3journal/jkag005)
Supplement: jkag005_Supplementary_Data [file jkag005_supplementary_data.zip › Supplemental_Figure_3_G3-2025-406427.pdf]

Supplemental Figure 3: Estimates of phenotypic correlations between stress hormone levels in hair from healthy pigs during the quarantine nursery phase and during the challenge nursery phase. CL = Cortisol, CN = Cortisone, DH = DHEA, DS = DHEA-S

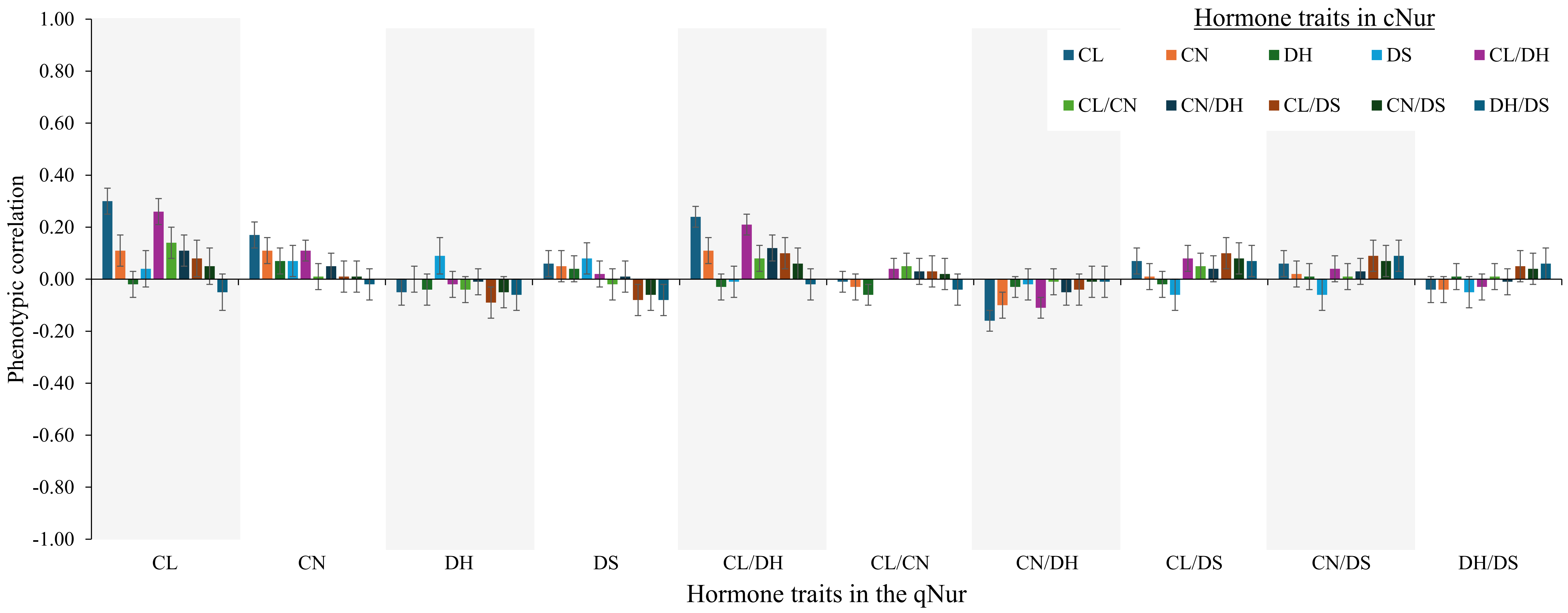
Hormone traits in cNur
CL
CN
DH
DS
CL/DH
CL/CN
CN/DH
CL/DS
CN/DS
DH/DS
Phenotypic correlation
1.00
0.80
0.60
0.40
0.20
0.00
-0.20
-0.40
-0.60
-0.80
-1.00
CL
CN
DH
DS
CL/DH
CL/CN
CN/DH
CL/DS
CN/DS
DH/DS
Hormone traits in the qNur
