# Supplementary material for: Genetics of retroactive measures of stress response in pigs before and after exposure to a disease challenge
Source: G3 (Bethesda). 2026 Jan 13;16(3):jkag005. doi: 10.1093/g3journal/jkag005 (PMC12958817; doi:10.1093/g3journal/jkag005)
Supplement: jkag005_Supplementary_Data [file jkag005_supplementary_data.zip › Supplemental_Figure_4_G3-2025-406427.pdf]

Supplemental Figure 4: Manhattan plot showing the 1 Mb windows explaining the percentage of genetic variance in cNur cortisol levels when they were adjusted for qNur cortisol levels as “baseline levels”.

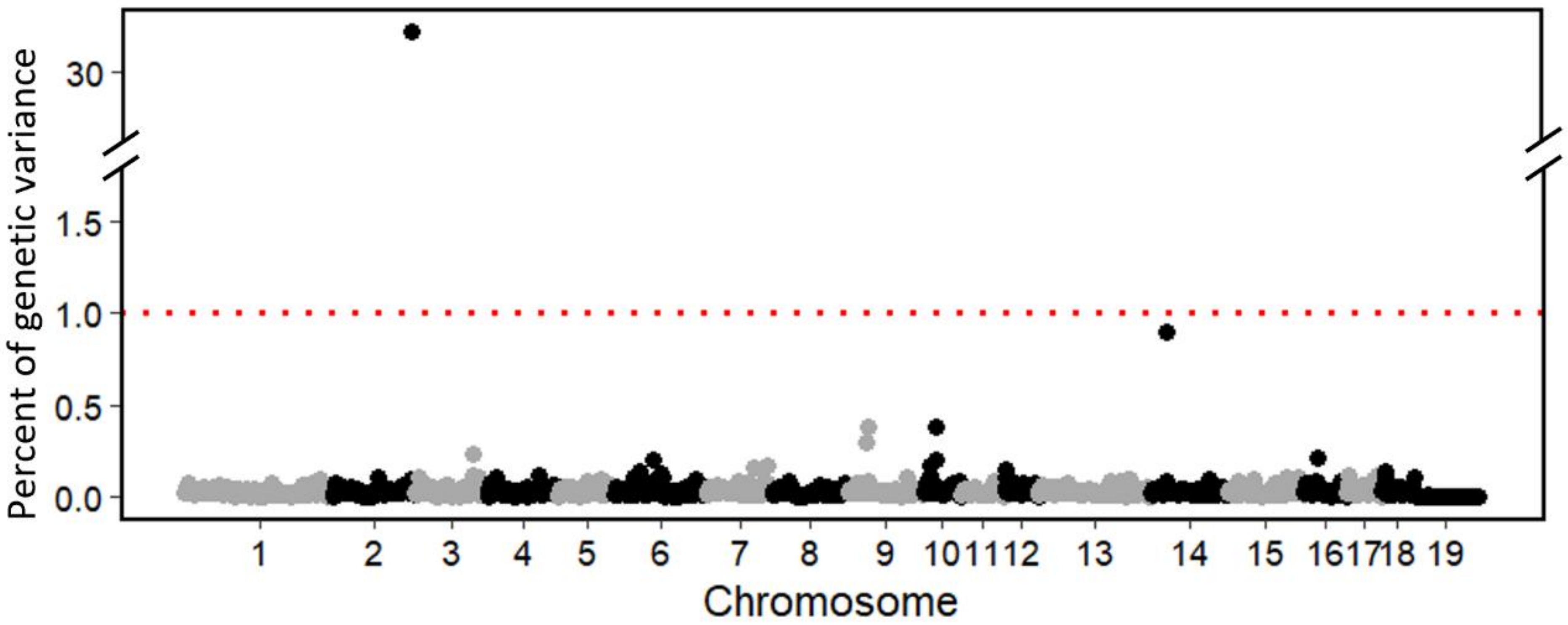
Percent of genetic variance
30
1.5
1.0
0.5
0.0
1
2
3
4
5
6
7
8
9
10
11
12
13
14
15
16
17
18
19
Chromosome
