# Supplementary material for: Genetics of retroactive measures of stress response in pigs before and after exposure to a disease challenge
Source: G3 (Bethesda). 2026 Jan 13;16(3):jkag005. doi: 10.1093/g3journal/jkag005 (PMC12958817; doi:10.1093/g3journal/jkag005)
Supplement: jkag005_Supplementary_Data [file jkag005_supplementary_data.zip › Supplemental_Figure_5_G3-2025-406427.pdf]

Supplemental Figure 5: Manhattan plots showing non-overlapping 0.25 Mb windows associated with pleiotropy between levels of stress hormones measured in hair under non-infectious stress and under infectious stress.

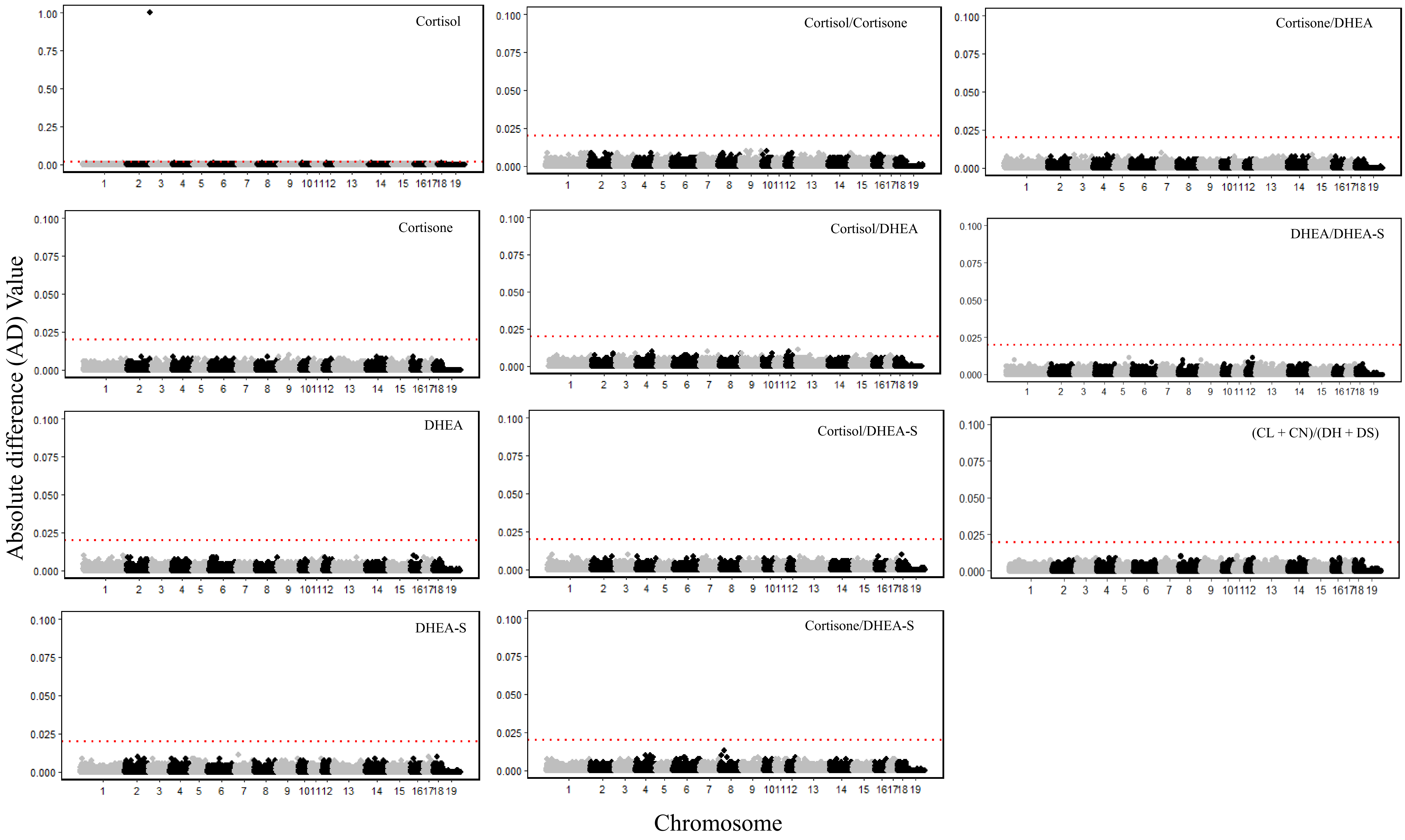

Absolute difference (AD) Value
Cortisol
Cortisol/Cortisone
Cortisone/DHEA
Cortisone
Cortisol/DHEA
DHEA/DHEA-S
DHEA
Cortisol/DHEA-S
(CL + CN)/(DH + DS)
DHEA-S
Cortisone/DHEA-S
Chromosome
