# Supplementary material for: Genetics of retroactive measures of stress response in pigs before and after exposure to a disease challenge
Source: G3 (Bethesda). 2026 Jan 13;16(3):jkag005. doi: 10.1093/g3journal/jkag005 (PMC12958817; doi:10.1093/g3journal/jkag005)
Supplement: jkag005_Supplementary_Data [file jkag005_supplementary_data.zip › Supplemental_Figure_6_G3-2025-406427.pdf]

Supplemental Figure 6: Manhattan plots showing non-overlapping 0.25 Mb windows associated with the pleiotropy between stress hormones measured in hair of pigs under non-infectious stress. Abbreviations: CL= cortisol, CN = cortisone, DH = DHEA, DS = DHEA-S) and backtest responses (VN = Vocalization number, VI = Vocalization intensity, SN = struggling number, SI = struggles intensity. The red line is an arbitrary threshold corresponding to the absolute difference (AD) value of 2%.

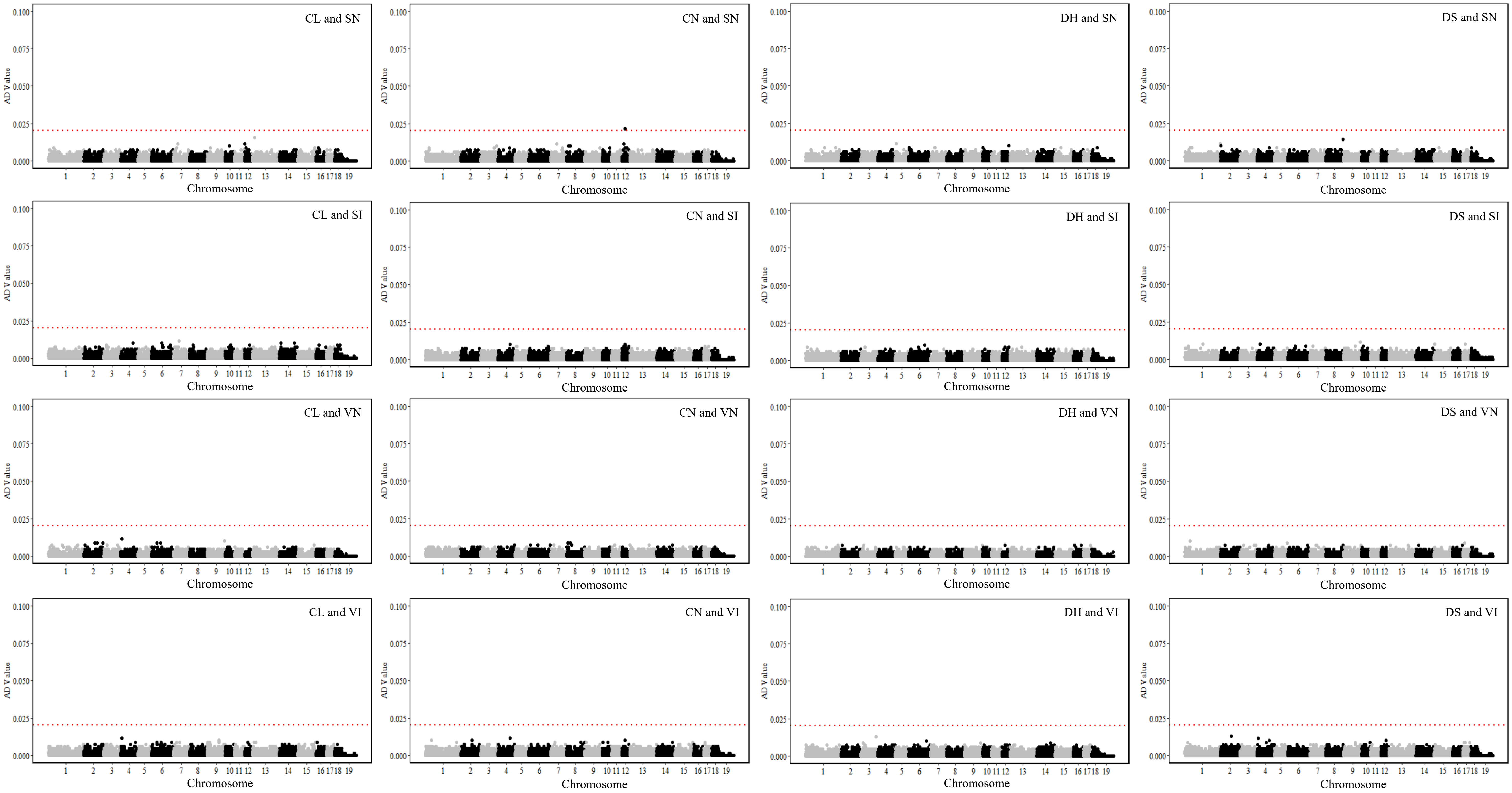
CL and SN
CN and SN
DH and SN
DS and SN
CL and SI
CN and SI
DH and SI
DS and SI
CL and VN
CN and VN
DH and VN
DS and VN
CL and VI
CN and VI
DH and VI
DS and VI
AD Value
0.100
0.075
0.050
0.025
0.000
1 2 3 4 5 6 7 8 9 10 11 12 13 14 15 16 17 18 19
Chromosome
