# Supplementary material for: Genetics of retroactive measures of stress response in pigs before and after exposure to a disease challenge
Source: G3 (Bethesda). 2026 Jan 13;16(3):jkag005. doi: 10.1093/g3journal/jkag005 (PMC12958817; doi:10.1093/g3journal/jkag005)
Supplement: jkag005_Supplementary_Data [file jkag005_supplementary_data.zip › Supplemental_Figure_Legends_G3-2025-406427.pdf]

List of supplemental figures

Supplemental Figure 1: Plots showing the relationship between the levels of stress hormones extracted from hair samples and the length of time the ground samples were stored before hormone extraction.

Supplemental Figure 2: Distributions of natural log-transformed stress hormone concentrations (pg/mg) and their ratios in hair regrowth during the challenge nursery phase at ~82 days of age.

Supplemental Figure 3: Estimates of phenotypic correlations between stress hormone levels in hair from healthy pigs during the quarantine nursery phase and during the challenge nursery phase. CL = Cortisol, CN = Cortisone, DH = DHEA, DS = DHEA-S

Supplemental Figure 4: Manhattan plot showing the 1 Mb windows explaining the percentage of genetic variance in cNur cortisol levels when they were adjusted for qNur cortisol levels as "baseline levels".

Supplemental Figure 5: Manhattan plots showing non-overlapping 0.25 Mb windows associated with pleiotropy between levels of stress hormones measured in hair under non-infectious stress and under infectious stress.

Supplemental Figure 6: Manhattan plots showing non-overlapping 0.25 Mb windows associated with the pleiotropy between stress hormones measured in hair of pigs under non-infectious stress. Abbreviations: CL= cortisol, CN = cortisone, DH = DHEA, DS = DHEA-S) and backtest responses (VN = Vocalization number, VI = Vocalization intensity, SN = struggling number, SI = struggles intensity. The red line is an arbitrary threshold corresponding to the absolute difference (AD) value of 2%.
