# Supplementary material for: Genetics of retroactive measures of stress response in pigs before and after exposure to a disease challenge
Source: G3 (Bethesda). 2026 Jan 13;16(3):jkag005. doi: 10.1093/g3journal/jkag005 (PMC12958817; doi:10.1093/g3journal/jkag005)
Supplement: jkag005_Supplementary_Data [file jkag005_supplementary_data.zip › Supplemental_Table_1_G3-2025-406427.docx]

**Supplemental Table 1**: Genetic parameters for stress hormone traits under IS and their genetic correlation estimates with hormone traits under NIS (off-diagonals) and backtest responses. Diagonal elements consist of the phenotypic variance and heritability estimate for hormone traits under IS, as well as their genetic correlation with the respective levels of the hormone under NIS.

|  |  | **Hormone traits under NIS** | | | | | | | | | | **Backtest responses** | | | |
| --- | --- | --- | --- | --- | --- | --- | --- | --- | --- | --- | --- | --- | --- | --- | --- |
|  | $\boldsymbol{\sigma}_{\boldsymbol{p}}^{\boldsymbol{2}}$  ${\hat{\boldsymbol{h}}}^{\boldsymbol{2}}$  ${\hat{\boldsymbol{r}}}_{\boldsymbol{g}}$ | **CL** | **CN** | **DH** | **DS** | **CL/DH** | **CL/CN** | **CN/DH** | **CL/DS** | **CN/DS** | **DH/DS** | **VN** | **SN** | **VI** | **SI** |
| **Hormone traits under IS** | **CL** | 0.16 (0.01)  0.23 (0.09)  **0.52 (0.20) ^b^** | 0.38  (0.42) | nc | 0.06  (0.26) | 0.29  (0.22) | nc | -0.03  (0.06) | 0.30  (0.21) ^c^ | 0.26  (0.23) ^c^ | 0.21  (0.23) | 0.26  (0.21) ^c^ | -0.01 (0.28) | 0.13  (0.22) | -0.18 (0.26) |
|  | **CN** | -0.04  (0.26) | 0.30 (0.02)  0.27 (0.10)  **0.11 (0.48)** | nc | -0.40  (0.29) ^c^ | nc | nc | 0.28  (0.47 | 0.28  (0.23) ^c^ | 0.40  (0.23) ^b^ | 0.20  (0.24) | 0.01  (0.21) | -0.48 (0.34) ^b^ | 0.01  (0.24) | -0.34 (0.28) ^c^ |
|  | **DH** | -0.74  (0.52) ^b^ | -0.79  (1.20) | 0.18 (0.01)  0.15 (0.09)  **nc** | -0.49  (0.58) | -0.43  (0.36) ^c^ | nc | -0.82  (1.01) | 0.17  (0.29) | 0.23  (0.31) | 0.39  (0.33) ^c^ | -0.08 (0.28) | -0.22 (0.43) | -0.45 (0.35) ^b^ | -0.10 (0.33) |
|  | **DS** | -0.02  (0.50) | 0.76  (0.54) ^c^ | nc | 1.23 (0.11)  0.01 (0.14)  **0.45 (0.41)** | -0.11  (0.39) | nc | 0.95  (0.93) | 0.02  (0.36) | 0.18  (0.39) | 0.03  (0.38) | -0.66 (0.64) ^c^ | -0.12 (0.52) | -0.90 (0.94) | 0.05  (0.48) |
|  | **CL/DH** | 0.94  (0.34) ^b^ | 0.46  (0.51) | nc | 0.21  (0.35) | 0.25 (0.02)  0.10 (0.09)  **0.60 (0.31) ^b^** | nc | 0.20  (0.53) | 0.24  (0.25) | 0.03  (0.28) | 0.02  (0.27) | 0.35  (0.27) ^b^ | -0.02 (0.33) | 0.53  (0.27) ^b^ | -0.13 (0.32) |
|  | **CL/CN** | 0.55  (0.24) ^b^ | 0.23  (0.45) | nc | 0.32  (0.28) ^c^ | nc | 0.22 (0.02)  0.20 (0.10)  **nc** | -0.26  (0.41) | -0.09  (0.23) | -0.27  (0.24) | -0.11  (0.23) | 0.45  (0.24) ^a^ | 0.63  (0.35) ^a^ | 0.32  (0.25) ^c^ | 0.43  (0.28) ^b^ |
|  | **CN/DH** | 0.10  (0.29) | 0.50  (0.57) | nc | -0.18  (0.30) | 0.40  (0.26) ^c^ | nc | 0.35 (0.03)  0.22 (0.11)  **0.44 (0.51)** | -0.22  (0.25) | 0.34  (0.27) ^c^ | -0.12  (0.26) | -0.70 (0.47) | -0.36 (0.35) ^c^ | 0.33  (0.24) ^c^ | -0.33 (0.29) ^c^ |
|  | **CL/DS** | 0.78  (0.93) | -0.12  (1.70) | -0.31  (0.67) | -0.14  (0.39) | 0.34  (0.40) | nc | -0.49  (1.13) | 1.14 (0.10)  0.00 (0.00)  **0.20 (0.37)** | 0.08  (0.40) | 0.04  (0.40) | 0.75  (0.57) ^b^ | 0.19  (0.72) | 0.75  (0.81) | -0.30 (0.53) |
|  | **CN/DS** | nc | nc | -0.36  (0.52) | -0.16  (0.32) | 0.23  (0.34) | nc | -0.51  (0.95) | 0.14  (0.31) | 1.23 (0.11)  0.00 (0.00)  **0.07 (0.33)** | 0.02  (0.32) | 0.28  (0.34) | -0.09 (0.72) | 0.52  (0.39) ^b^ | -0.41 (0.57) |
|  | **DH/DS** | 0.34  (1.27) | -0.92  (1.18) | 0.18  (0.84) | -0.10  (0.34) | -0.12  (0.42) | nc | -0.69  (0.88) | 0.05  (0.33) | -0.09  (0.35) | 0.03 (0.08)  0.03 (0.16)  **0.06 (0.35)** | 0.36  (0.45) | 0.02  (0.59) | 0.00  (0.00) | -0.22 (0.49) |

nc = Did not converge; Estimate is significantly different from zero: ^a^ = p ≤ 0.05, ^b^ = (p ≤ 0.15), ^c^ = (p ≤ 0.25)

^†^ = Estimate is not significantly different from one at p ≤ 0.05. CL = Cortisol, CN = Cortisone, DH = DHEA, DS = DHEA-S,
