# Supplementary material for: Genetics of retroactive measures of stress response in pigs before and after exposure to a disease challenge
Source: G3 (Bethesda). 2026 Jan 13;16(3):jkag005. doi: 10.1093/g3journal/jkag005 (PMC12958817; doi:10.1093/g3journal/jkag005)
Supplement: jkag005_Supplementary_Data [file jkag005_supplementary_data.zip › Supplemental_Table_2_G3-2025-406427.docx]

**Supplemental Table 2:** Estimates of phenotypic correlations between responses to the 30 s backtest performed on young healthy pigs in the quarantine nursery and log-transformed hormone levels measured in hair growth during the challenge nursery during a polymicrobial disease challenge.

|  | Struggle intensity | Struggle number | Vocalization intensity | Vocalization number |
| --- | --- | --- | --- | --- |
| CL | 0.03 (0.04) | 0.01 (0.04) | 0.01 (0.04) | 0.02 (0.04) |
| CN | 0.01 (0.05) | 0.04 (0.05) | 0.03 (0.05) | 0.01 (0.05) |
| DH | 0.02 (0.04) | -0.04 (0.04) | 0.01 (0.04) | 0.02 (0.04) |
| DS | 0.01 (0.06) | 0.00 (0.06) | -0.03 (0.06) | -0.04 (0.06) |
| CL + CN (SOG) | 0.02 (0.05) | 0.01 (0.05) | 0.07 (0.05) | 0.05 (0.05) |
| DH + DS (SOD) | -0.04 (0.06) | -0.13 (0.06) | -0.07 (0.06) | -0.10 (0.06) |
| CL/CN | 0.03 (0.05) | -0.02 (0.05) | 0.04 (0.05) | 0.08 (0.05) |
| CL/DH | 0.00 (0.04) | 0.04 (0.04) | 0.00 (0.04) | 0.01 (0.04) |
| CN/DH | 0.00 (0.05) | 0.06 (0.05) | 0.03 (0.06) | -0.11 (0.06) |
| CL/DS | 0.00 (0.06) | 0.00 (0.06) | 0.04 (0.06) | 0.06 (0.06) |
| CN/DS | -0.02 (0.06) | 0.01 (0.06) | 0.02 (0.06) | 0.02 (0.06) |
| DH/DS | -0.04 (0.06) | -0.06 (0.06) | 0.00 (0.06) | 0.00 (0.06) |
| SOG/SOD | 0.01 (0.06) | 0.08 (0.06) | 0.08 (0.06) | 0.09 (0.06) |
| SOG/DH | 0.01 (0.05) | 0.05 (0.05) | 0.07 (0.05) | 0.06 (0.05) |

CL = Cortisol, CN = Cortisone, DH = DHEA, DS = DHEA-S, SOG = sum of glucocorticoids, SOD = sum of DHEA(S)
