# Supplementary material for: Genetics of retroactive measures of stress response in pigs before and after exposure to a disease challenge
Source: G3 (Bethesda). 2026 Jan 13;16(3):jkag005. doi: 10.1093/g3journal/jkag005 (PMC12958817; doi:10.1093/g3journal/jkag005)
Supplement: jkag005_Supplementary_Data [file jkag005_supplementary_data.zip › Supplemental_Table_3_G3-2025-406427.docx]

**Supplemental Table 3:** Estimated effects (in phenotypic standard deviation units) on the natural log-scale for an extra copy of the minor allele at the imputed lead SNP (rs335962816) on the natural log-transformed hormone traits in hair regrown during the challenge nursery phase of the natural disease challenge model, as well as the variability of the estimates across all companies (A, C, D, E, and F) and the heritability estimates of traits when the lead SNP genotypes were fitted as a covariate in the GBLUP model (h^2^-adj*).

| ln(Hormone traits) | Main effect | p-value | h^2^-adj* (SE) | Effect_A | Effect_C | Effect_D | Effect_E | Effect_F | p-value |
| --- | --- | --- | --- | --- | --- | --- | --- | --- | --- |
| CL | -0.98 | < 0.001 | 0.20 (0.09) | -0.86 | -0.36 | -1.63 | -0.93 | -0.93 | < 0.05 |
| CN | -0.47 | < 0.001 | 0.29 (0.11) | -0.18 | -0.28 | -1.06 | -0.38 | 0.01 | < 0.05 |
| DH | -0.06 | 0.58 | 0.15 (0.10) | -0.17 | 0.19 | -0.74 | 0.08 | 0.65 | 0.41 |
| DS | 0.16 | 0.22 | 0.00 (0.13) | 1.61 | 0.59 | 0.00 | 0.07 | 0.07 | 0.44 |
| CL + CN (SOG) | -0.77 | < 0.001 | 0.25 (0.11) | -1.01 | -0.60 | -2.67 | -1.17 | -0.88 | < 0.05 |
| DH + DS (SOD) | 0.07 | 0.59 | 0.12 (0.14) | 0.59 | 0.55 | -0.56 | 0.33 | -0.15 | 0.49 |
| CL/CN | -0.23 | 0.04 | 0.23 (0.10) | -0.97 | 0.04 | -0.17 | -0.37 | -1.43 | 0.44 |
| CL/DH | -0.71 | < 0.001 | 0.03 (0.08) | -0.61 | -0.37 | -0.83 | -0.79 | -1.03 | 0.60 |
| CN/DH | -0.50 | < 0.001 | 0.00 | -1.15 | -0.51 | -0.54 | -0.34 | -0.37 | 0.61 |
| CL/DS | -0.42 | < 0.001 | 0.24 (0.11) | -0.17 | -0.24 | -0.67 | -0.47 | -0.22 | 0.64 |
| CN/DS | -0.38 | 0.06 | 0.00 | -0.92 | -0.40 | -0.51 | -0.23 | -0.02 | 0.62 |
| DH/DS | -0.19 | 0.15 | 0.02 (0.13) | -0.91 | -0.31 | -0.14 | 0.04 | -0.02 | 0.25 |
| SOG/SOD | -1.70 | < 0.001 | 0.00 | -0.77 | -1.38 | -2.30 | -1.81 | -1.08 | 0.70 |
| SOG/DH | -1.59 | < 0.001 | 0.16 (0.12) | -0.98 | -1.09 | -2.06 | -1.72 | -1.72 | 0.67 |

CL = Cortisol, CN = Cortisone, DH = DHEA, DS = DHEA-S, SOG = sum of glucocorticoids, SOD = sum of DHEA(S)
