# Supplementary material for: Genetics of retroactive measures of stress response in pigs before and after exposure to a disease challenge
Source: G3 (Bethesda). 2026 Jan 13;16(3):jkag005. doi: 10.1093/g3journal/jkag005 (PMC12958817; doi:10.1093/g3journal/jkag005)
Supplement: jkag005_Supplementary_Data [file jkag005_supplementary_data.zip › Supplemental_Table_Legends_G3-2025-406427.pdf]

List of supplemental tables

Supplemental Table 1: Genetic parameters for stress hormone traits under IS and their genetic correlation estimates with hormone traits under NIS (off-diagonals) and backtest responses. Diagonal elements consist of the phenotypic variance and heritability estimate for hormone traits under IS, as well as their genetic correlation with the respective levels of the hormone under NIS.

Supplemental Table 2: Estimates of phenotypic correlations between responses to the 30 s backtest performed on young healthy pigs in the quarantine nursery and log-transformed hormone levels measured in hair growth during the challenge nursery during a polymicrobial disease challenge.

Supplemental Table 3: Estimated effects (in phenotypic standard deviation units) on the natural log-scale for an extra copy of the minor allele at the imputed lead SNP (rs335962816) on the natural log-transformed hormone traits in hair regrown during the challenge nursery phase of the natural disease challenge model, as well as the variability of the estimates across all companies (A, C, D, E, and F) and the heritability estimates of traits when the lead SNP genotypes were fitted as a covariate in the GBLUP model (h^2-adj*).

Supplemental Table 4: Gene ontology terms enriched among 0.25 Mb windows for levels of cortisol, cortisone, DHEA, and DHEA-S in hair of pigs exposed to a natural polymicrobial disease challenge based on gene set enrichment analyses.

Supplemental Table 5: Gene ontology terms enriched among 0.25 Mb genomic windows that explained different percentages of the genetic variance for levels of cortisol, cortisone, DHEA, and DHEA-S in hair of pigs before and after they were exposed to a natural polymicrobial disease challenge.
