# Supplementary material for: Genetics of retroactive measures of stress response in pigs before and after exposure to a disease challenge
Source: G3 (Bethesda). 2026 Jan 13;16(3):jkag005. doi: 10.1093/g3journal/jkag005 (PMC12958817; doi:10.1093/g3journal/jkag005)
Supplement: jkag005_Supplementary_Data [file jkag005_supplementary_data.zip › Supplementary_Data_Access_Procedure_G3-2025-406427.pdf]

Procedure for requesting access to the full data

Requests for access to the data should be made in writing to the corresponding author and must include the names of the individuals and of their respective employers who will be provided access to the data, the objectives of the research that will be conducted using the data, details on how the results generated by the proposed research will be used, details on what information will be made public in any materials/publications that will be generated from the proposed research, details on how confidentiality of the data that is requested will be guaranteed, and details on all aspects of any intellectual property that may result from the proposed research.

Parties responsible for evaluating requests

Granting parties: the corresponding author (J. Dekkers), the project director of the original research project that generated the data (M. Dyck), and the President of PigGen Canada.

Criteria and procedure used to evaluate such requests

The corresponding author will forward the request to the other two parties, who will each evaluate the request based on overlap of the proposed research with ongoing research using the same data by the project director and co-directors and based on the commercial interests of the PigGen Canada investing members and provide a written recommendation on approval or disapproval of the request, along with the reasons in the case of disapproval, within 45 days of the request being made. Each party may ask for further clarification on the request before providing a recommendation. In the case of disagreement among the responsible parties, a meeting will be convened to discuss and obtain a consensus decision. If a consensus decision cannot be reached, the final decision will rest with M. Dyck. Expected response time to requests for data access is 60 days.

Conditions for gaining access to the data

1. The requesting party must execute a material transfer agreement provided by the granting parties.
2. Confidentiality of the data provided must be guaranteed, and all liability resulting thereof, by the requesting party in writing, as part of the material transfer agreement.
3. Results of the research and any public materials/publications that will be released must be shared with the corresponding author for review by the granting parties prior to publication, for which they will have 45 days. The requesting party will accommodate all reasonable changes to the materials/publications that are consistent with the conditions outlined herein.
4. Results of the proposed research will not be used nor licensed for commercial purposes.
5. Any intellectual property that is generated by the proposed research will be held jointly by the requesting party and the members of the original research project.
6. The requesting party cannot sign any agreement regarding sharing of the data without the written agreement of the granting parties

Usage or types of requests would lead to a denial of access

i. The proposed research overlaps with ongoing research by the project director or co-directors of the original project or with other third-party requests that have been granted.
ii. Outcomes of the proposed research will be used, patented, or licensed for commercial or university interests by the third party and/or its associates/funders.
iii. The proposed research and the results that will be made available from it compromise the commercial interests of the investing members of PigGen Canada.
iv. The third party is not able to guarantee confidentiality of the data that is requested.
